# *N*-Heterocyclic Carbene Platinum(IV) as Metallodrug Candidates: Synthesis and ^195^Pt NMR Chemical Shift Trend

**DOI:** 10.3390/molecules25143148

**Published:** 2020-07-09

**Authors:** Mathilde Bouché, Bruno Vincent, Thierry Achard, Stéphane Bellemin-Laponnaz

**Affiliations:** 1 Institut de Physique et Chimie des Matériaux de Strasbourg, Université de Strasbourg-CNRS UMR7504, 23 rue du Loess, BP 43 CEDEX 2, 67034 Strasbourg, France; mathilde.bouche9@gmail.com (M.B.); thierry.achard@ipcms.unistra.fr (T.A.); 2 Service de RMN, Fédération de Chimie Le Bel, Université de Strasbourg, CNRS FR2010, 1 rue Blaise Pascal, BP 296R8, CEDEX, 67008 Strasbourg, France; bvincent@unistra.fr

**Keywords:** *N*-heterocyclic carbene, platinum, metal complexes, ^195^Pt NMR

## Abstract

A series of octahedral platinum(IV) complexes functionalized with both *N*-heterocyclic carbene (NHC) ligands were synthesized according to a straightforward procedure and characterized. The coordination sphere around the metal was varied, investigating the influence of the substituted NHC and the amine ligand in trans position to the NHC. The influence of those structural variations on the chemical shift of the platinum center were evaluated by ^195^Pt NMR. This spectroscopy provided more insights on the impact of the structural changes on the electronic density at the platinum center. Investigation of the in vitro cytotoxicities of representative complexes were carried on three cancer cell lines and showed IC_50_ values down to the low micromolar range that compare favorably with the benchmark cisplatin or their platinum(II) counterparts bearing NHC ligands.

## 1. Introduction

In the treatment of solid tumors, platinum-based chemotherapy remains a top-notch drug thanks to its high anticancer efficiency and high remission rates in selected cancers, up to 90% in patients suffering testicular cancer [1]. However, the severe systemic toxicity and poor selectivity for tumors, with only 1% of the injected dose of cisplatin actually reaching their target, stress the importance to explore new strategies to stabilize the platinum center and prevent off-target interactions [2]. Lot of efforts have focused on adjusting the coordination sphere of the platinum or oxidizing the well established Pt(II) center into redox-activable Pt(IV) pro-drugs [3,4,5]. In particular, platinum complexes functionalized with *N*-heterocyclic carbenes have appeared as promising alternatives to cisplatin, as possible chemotherapeutics targeting the mitochondria [6,7,8,9]. In addition to investigating the redox potential of Pt(IV) complexes, ^195^Pt NMR is a valuable technique for the fast and standardized characterization of platinum complexes to complement routine characterization [10,11,12,13,14,15]. Moreover, investigating the chemical shift in ^195^Pt NMR is a prerequisite to further enable mechanistic and pharmacokinetic investigations of the platinum complex and its metabolites using ^195^Pt spectroscopy [16], as an alternative to LA-ICP-MS [17] or X-ray-based techniques [18,19,20,21]. Of note, Huynh et al. recently suggested the direct correlation of the platinum chemical shift in ^195^Pt NMR to both the electronic density at the platinum center and the electronic donation of the coordination sphere, in particular in the case of *N*-heterocyclic carbene (NHC)-platinum complexes [22,23,24]. Moreover, a linear correlation of the ^195^Pt NMR chemical shift with the in vitro anticancer activity (IC_50_) has been noted in azido-Pt(IV) complexes [25]. The chemistry in the solution of cisplatin and its derivatives have been studied by ^195^Pt NMR spectroscopy. In particular, they have been used to characterize related complexes with aqua, chloro, nitrato, sulfato, acetate, and phosphate ligands [26,27]. Therefore, we report herein a series of NHC-Pt(IV) complexes and a few examples of their Pt(II) metabolites possibly formed in vitro, that were synthesized and characterized using routine techniques. In vitro activities against three cancer cell lines of representative NHC-Pt(IV) complexes are also presented. Moreover, the shift in the platinum resonance signal in ^195^Pt NMR is investigated and discussed as a function of tuning their oxidation degree and coordination sphere.

## 2. Results and Discussion

### 2.1. Synthesis of the Platinum(II) and Platinum(IV) Complexes

**Scheme 1:**
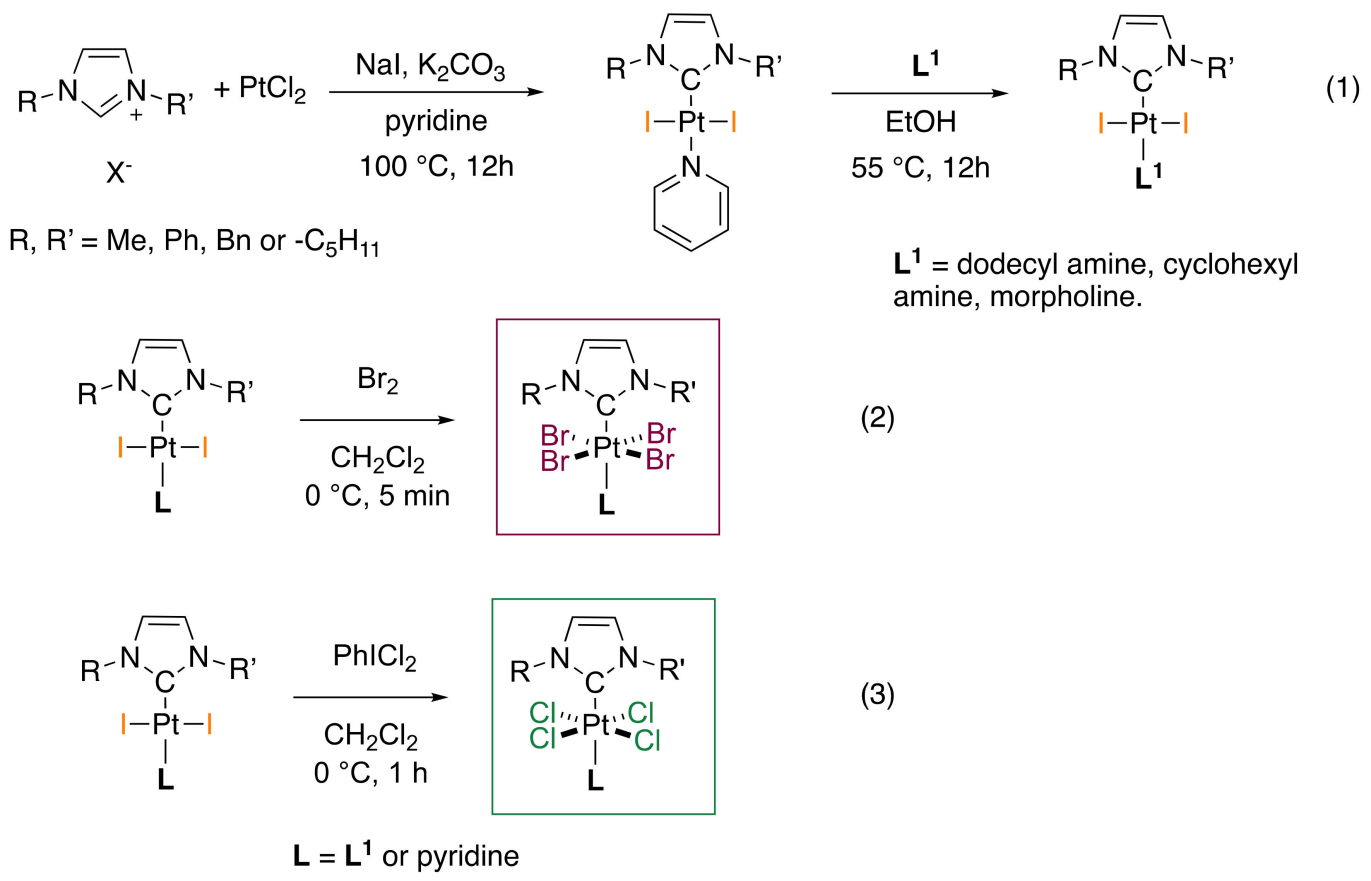
General synthesis of the platinum (II) and platinum (IV) complexes.

All NHC-Pt complexes were prepared using standard synthetic procedures as previously reported. The general scheme for the synthesis of the Pt(II) and Pt(VI) complexes is described in Scheme 1. First, platinum(II) NHC pyridine complexes were synthesized involving the in situ deprotonation of the imidazolium salt with K_2_CO_3_ and the coordination of the carbene to the PtCl_2_ precursor in dry amine with excess NaI overnight ((1), first step, Scheme 1) [28]. Chemical variation was then possible by the ligand substitution of the pyridine with various nitrogen-based ligands as shown in (1), second step, Scheme 1. The obtained (NHC)PtI_2_(pyridine) and (NHC)PtI_2_(amine) complexes could further be oxidized according to a procedure previously reported by us [9]. The aforementioned Pt(II) complexes were reacted with a 10-fold excess of bromine at 0 °C to obtain the corresponding (NHC)PtBr_4_(L) complexes ((2), Scheme 1). The reaction proceeded very quickly and cleanly to give the expected corresponding Pt(IV) species after only 5 min of reaction. The chlorinated complexes ((NHC)PtCl_4_L) were obtained by direct oxidation using a 2-fold excess of freshly prepared hypervalent iodine reagent PhICl_2_ ((3), Scheme 1). The reaction was complete after 1 h at 0 °C. All the platinum(IV) complexes were easily isolated by precipitation with pentane. They were usually obtained in high chemical yield and were stable under air in the solid state or in chlorinated solvents and showed increasing solubility in organic solvents in respect to the length of alkyl chains on the NHC or amine ligand.

**Scheme 2:**
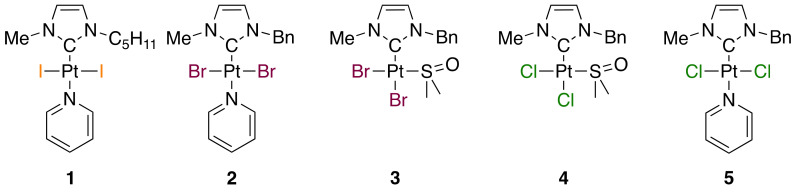
Molecular structure of the *N*-heterocyclic carbene (NHC)-Pt(II) references.

Scheme 2 displays the molecular structures of the five platinum(II) NHC complexes used either as precursors for the Pt(IV) syntheses, or as a reference for the studies discussed here. The (NHC)Pt(II)(DMSO) complexes **3** and **4** were obtained by a transmetallation route from the bis(benzyl)imidazol-2-ylidene silver(I) bromide precursor reacted with platinum salt as previously published by us [7]. The NHC Pt(II) complexes **2** and **5** were obtained using the procedure described in (1), Scheme 1 using the corresponding salt NaBr or NaCl respectively.

**Scheme 3:**
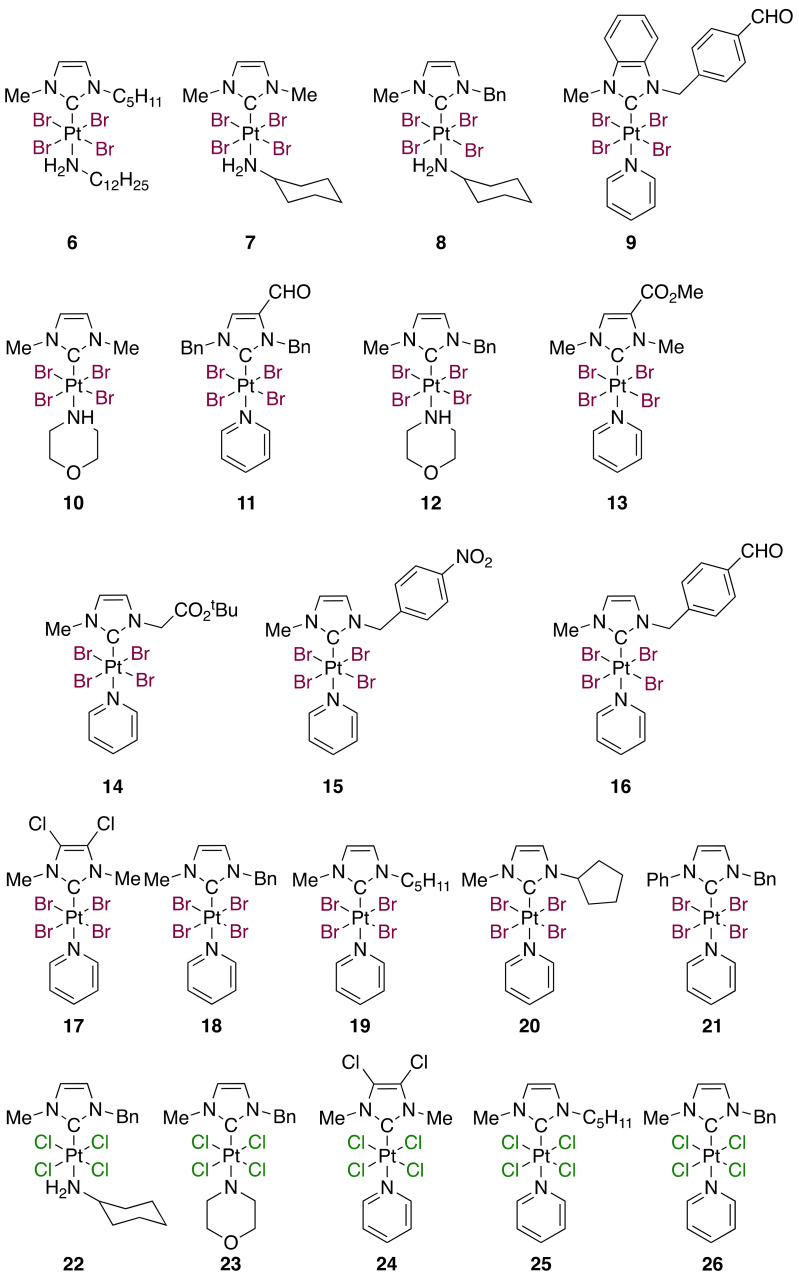
Molecular structure of the NHC-Pt(IV) complexes.

Scheme 3 displays all the platinum(IV) that were synthesized and characterized. A series of (NHC)PtBr_4_(amine) complexes bearing a (methyl-, benzyl-)NHC, were obtained in a 99% yield with various trans amine ligands, i.e., dodecylamine, cyclohexylamine, morpholine and pyridine, corresponding to complexes **6**, **8**, **12** and **18,** respectively. Identically, the (NHC)PtCl_4_(amine) complexes with varying amine ligands were obtained in good yields, the corresponding amine ligand being a cyclohexylamine for **22**, a morpholine for **23** and a pyridine for **26**. The versatile synthesis tolerated the NHC structural variations among the (NHC)PtBr_4_(amine) family, with *N*-substituents being a CH_2_-*tert*-butylacetate for **14**, *p*-nitro-benzyl for **15**, *p*-benzaldehyde for **16**, a pentyl for **19**, a cyclopentyl for **20** and a phenyl for **21**, all obtained in 99% yield. The functionalization of the positions 4 and 5 of the NHC ligand did not hamper the oxidation reaction, and the (NHC)PtBr_4_(amine) complexes **9**, **11**, **13** and **17** were isolated in high yield, corresponding respectively to a benzimidazole, 4-metyl- and 5-aldehyde, 4-methylester, and 4,5-dichloro-NHC. Similarly, the (NHC)PtCl_4_(amine) complexes **24** bearing a 4,5-dichloro-NHC and **25** functionalized with a pentyl-*N*-substituted NHC were obtained also in a yield up to 99%. The characterization by the ^1^H NMR showed that all the proton signals displayed a shift to a lower field compared to their imidazolium precursors which proved typical for such complexes. Overall, the NHC-Pt(IV) complexes showed a signal duplication typically observed for all the protons in up to the ^5^J position to the platinum center, suggesting an enhanced coupling with the ^195^Pt isotope compared to their NHC-Pt(II) precursors. Of note, the very low solubility of (NHC)PtBr_4_(pyridine) complexes prevented the successful acquisition of the ^13^C NMR of complexes **4**, **5**, **11**, **20** and **26**, or rendered the carbenic carbon signal not visible. However, in the case of more lipophilic complexes, coupling between the carbenic carbon and the platinum center was observed in ^13^C NMR. Such a trend was found typical throughout all the NHC-Pt(IV) complexes, the carbenic carbon signal appearing as a singlet and doublet system, possibly due to the heavy atom effect of platinum [29]. Moreover, chemical shifts to a higher field of the carbenic carbon were also observed by ^13^C NMR spectroscopy, ca. δ 109–120 ppm in the case of NHC-Pt(IV) complexes, while (NHC)PtI_2_(amine) complexes previously reported by us [30,31] and others [32,33] typically show a signal shift at least 30 ppm greater.

**Figure 1:**
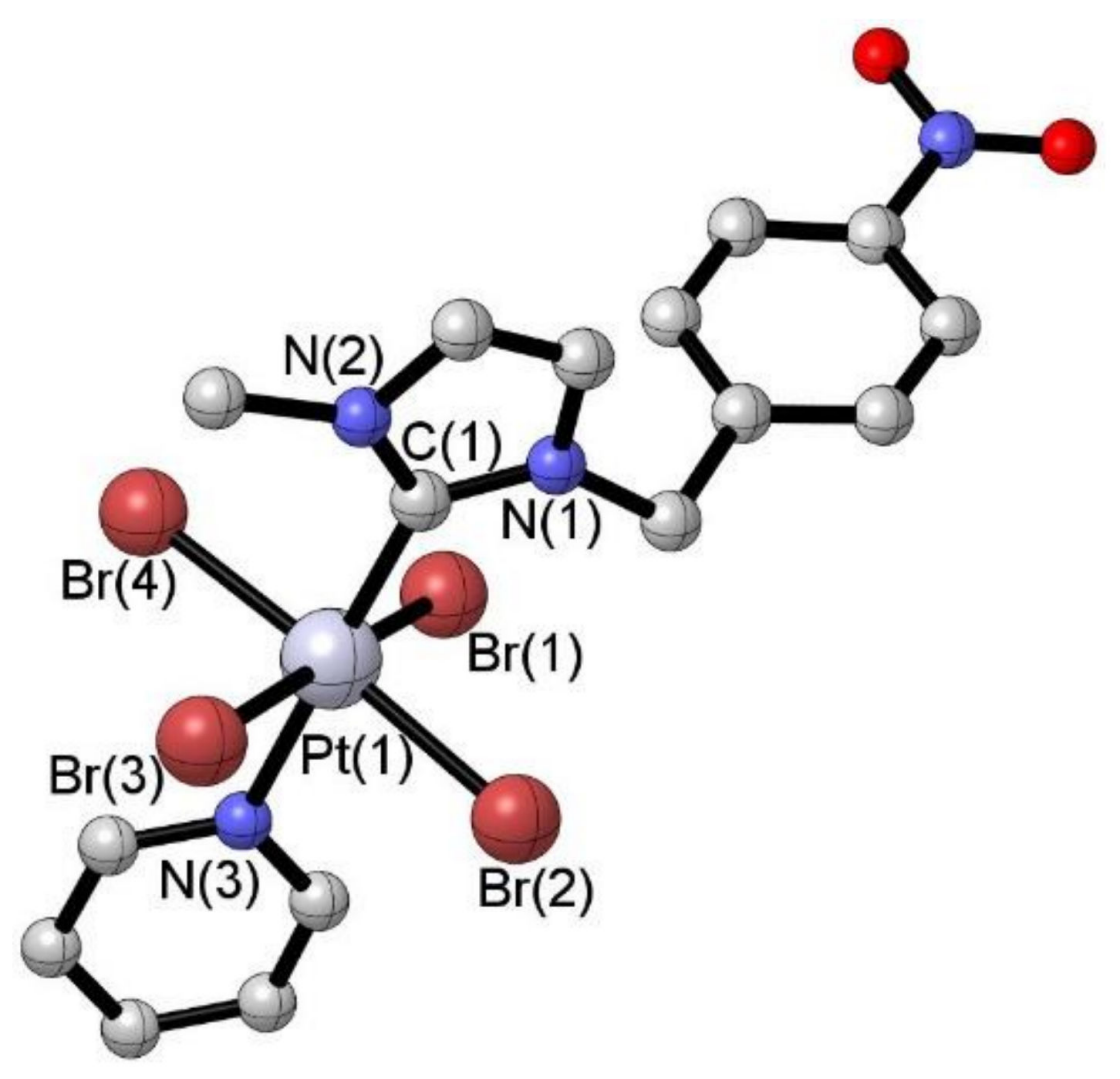
Molecular structure of complex **15**. Selected bond distances (Å) and angles (deg): C(1)-Pt(1), 2.057(8); Br(1)-Pt(1), 2.4882(8); Br(2)-Pt(1), 2.4657(8); Br(3)-Pt(1), 2.4615(8); Br(4)-Pt, 2.4839(8); N(3)-Pt(1), 2.128(6); C(1)-Pt(1)-N(3), 179.2(3); C(1)-Pt(1)-Br(3), 92.9(2); N(3)-Pt(1)-Br(3), 87.10(16); Br(2)-Pt(1)-Br(3), 86.10(3); Br(1)-Pt(1)-Br(4), 177.05(3).

Among these Pt(IV) complexes, the molecular structure of the (NHC)PtBr_4_(amine) complex **15** was determined by X-ray diffraction and is presented in Figure 1. The platinum center shows an octahedral geometry with bromine ligands forming a distorted square planar shape in equatorial position, comparable to other (NHC)PtBr_4_(amine) complexes previously reported by us [6,7]. The pyridine ligand is located in trans position to the NHC with a platinum-pyridine length of 2.128(6) Å while the NHC-platinum bond is found to be 2.057(8) Å. The molecular structure of the (NHC)PtCl_4_(amine) complex **23** revealed a comparable geometry with overall shorter bonds between the platinum center and the ligands, reflective of the influence of the coordination sphere on platinum’s electronic density, exemplified by the NHC-platinum length of 2.034(3) Å, and the chloride-platinum bonds in the range of 2.327(3)–2.336(3) Å [7].

### 2.2. In Vitro Activities against Cancer Cell Lines

Among the series of NHC-platinum complexes herein, a series of the most soluble complexes were selected for the evaluation of their in vitro anticancer activities. Overall, most NHC-Pt(IV) complexes were found to display comparable IC_50_ values to cisplatin in the range of 0.5–23 µM. Contrastingly, the complex **16** showed disparate anticancer activities depending on the cancer cell line with the IC_50_ values of 5.42 µM and 81.09 µM against the PC3 or HCT116 respectively (Table 1). Of note, the low solubility of this complex in aqueous media might explain the low IC_50_ values observed in this study. The series of the (NHC)PtBr_4_(amine) complexes **6**, **8**, **12** and **19** show potencies that compare favorably with the NHC-Pt(II) complexes which are expected to be the species released upon their redox activation. Such a result is in line with our previous findings suggesting their rapid reduction and release of the active species [6,7]. Remarkably, the (NHC)PtCl_4_(amine) complexes **22** and **25** show the most promising in vitro potencies with IC_50_ values in the low micromolar range against the three tested cancer cell lines.

### 2.3. ^195^Pt NMR Spectroscopy

The NHC-platinum complexes were further characterized using a ^1^H detection inverse NMR spectroscopy sequence which was preferred to direct the ^195^Pt measurement in regard of shorter acquisition time and enhanced sensibility. This was supported by a test experiment using complex **8** as a reference, comparing spectra obtained in direct ^195^Pt NMR or indirect HMQC ^1^H-^195^Pt NMR, and both showed a signal peak at δ_Pt_ = −2168 ppm irrespective of the sequence used. Table 2 displays the ^195^Pt chemical shift NMR of all the complexes and carbenic carbon signal in the ^13^C NMR, when observed. The most significant variation in the platinum chemical shift was found as a function of the oxidation state of the platinum center. All the (NHC)PtBr_4_(amine) complexes **6**–**21** displayed a platinum chemical shift in the range of δ_Pt_ −1901 to −2196 ppm while the (NHC)PtCl_4_(amine) complexes **22**–**26** were observed at δ_Pt_ −883 to −795 ppm and all other NHC-Pt(II) complexes **1**–**5** displayed a chemical shift below −3304 ppm.

Of note, the use of ^1^H detection inverse spectroscopy proved of high interest for most complexes to observe the ^4^*J*_H-Pt_ long-range couplings between the platinum center and C_3_, C_4_ protons on the NHC backbone as well as the protons on the *N*-substituents of the NHC. This strong chemical coupling suggests a high electronic delocalization from the platinum center to the substituents of the NHC ligand which yet does not seem to significantly affect the chemical shift in ^195^Pt NMR. Thus, the series of NHC-Pt(IV) complexes **14**–**16**, **19** and **20** show a platinum chemical shift decrease from δ_Pt_ = −2032 to −2070 ppm with the *N*-substituents following the trend Cy > C_5_H_11_ > Bn > CH_2_CO_2_^t^Bu. Similarly, the functionalization of C_3_ and C_4_ positions on the NHC backbone of the NHC-Pt(IV) complexes is shown to have a negligible effect on the platinum shift with Δδ_Pt_ = 2 ppm between complexes **13** and **11**. Moreover, a large platinum chemical shift variation Δδ_Pt_ = 64 ppm was observed between the imidazolin-2-ylidene ligand in **16** (δ_Pt_ −2063 ppm) and the benzimidazolin-2-ylidene ligand in **9** (δ_Pt_ −2127 ppm), which was found to correlate with the Δδ_C_ = 23.1 ppm of their carbenic carbon observed by ^13^C NMR. Among the series of the NHC-Pt(IV) complexes, the variation of the trans amine ligand shows a trend in the platinum chemical shift that follows the amine’s basicity from δ_Pt_ −2040 ppm for complex **18** to δ_Pt_ −2196 ppm for complex **6**. Thus, the trend in the platinum chemical shift is found to be **18** > **12** > **8** > **6**, corresponding to a trans ligand being pyridine > morpholine > cyclohexylamine > dodecylamine. Of note, the same trend is visible while comparing their carbenic carbon shift as complex **18** bearing a pyridine shows a δ_C_ of 109.3 ppm while its cyclohexylamine counterpart **8** shows a shift up to 115.2 ppm. Moreover, the (NHC)PtCl_4_(amine) complexes follow the same trend with platinum chemical shifts being **26** > **23** > **22**, corresponding to the trans amine ligand pyridine > morpholine > cyclohexylamine.

## 3. Materials and Methods

All the manipulations of the air- and moisture-sensitive compounds were carried out using standard Schlenk techniques under an argon atmosphere and the solvents were purified and degassed following standard procedures. All the reagents were purchased from commercial chemical suppliers (Acros (Illkirch, France), Alfa Aesar (Lancashire, UK), and TCI Europe (Paris, France)) and used without further purification. ^1^H and ^13^C nuclear magnetic resonance (NMR) spectra were recorded on a Brucker AVANCE 300 or Bruker AVANCE 500 spectrometer (Bruker, Wissembourg, France) using the residual solvent peak as a reference (CDCl_3_: δ_H_ = 7.26 ppm; δ_C_ = 77.16 ppm) at 295 K. The HMQC ^1^H-^195^Pt spectra were recorded on a Bruker AVANCE 600 spectrometer using the residual solvent peak as reference for the ^1^H calibration and an external reference for the ^195^Pt (H_2_PtCl_6_ in D_2_O: δ_Pt_ = 0 ppm) at the Institut de Chimie NMR Facility of the University of Strasbourg. Positive mode electrospray ionization mass spectra (ESI-HRMS) analyses were carried out on microTOF, Bruker Daltonics (Bruker, Wissembourg, France).

All the syntheses and characterizations are available in the Supplementary Materials.

## 4. Conclusions

In the present work, a series of *N*-heterocyclic carbene-coordinated platinum(IV) complexes were synthesized in high yield according to a versatile procedure. All the complexes were found stable in the air and in chlorinated solvents for months. Some representative examples of these NHC-Pt(IV) complexes were selected for the in vitro evaluation of their cancer inhibitory properties and compared to their possible Pt(II) metabolites formed in the biological environment. Overall, the lipophilic (NHC)PtCl_4_(amine) complex **22** was found to induce the greater in vitro potencies toward selected cancer cell lines with IC_50_ values in the low micromolar range.

In the development of platinum-based metallodrugs, numerous parameters have to be considered in addition to the apparent electronic density at the platinum center that may be reflected by the ^195^Pt NMR chemical shift, namely lipophilicity and pharmacological properties and so forth. Moreover, the balance between the stability of the platinum drugs in the blood stream and their ability to form metabolites and interact with DNA is difficult to anticipate by finetuning the coordination sphere of the platinum. However, the ^195^Pt NMR has proved to be a helpful probe in investigating the biological activity of platinum-based drugs. For example, a recent study involving the monitoring of carboplatin after subcutaneous injection in rats was studied using ^195^Pt NMR [34]. Thus, all the complexes presented here were characterized with standard techniques and the influence of structural variations, i.e., on one hand the coordination sphere and on the other hand the NHC ligand’s functionalization, were correlated to their chemical shift in ^195^Pt NMR. All the (NHC)PtBr_4_(amine) complexes displayed platinum chemical shifts in the range of δ_Pt_ −1900 to −2200 ppm while the (NHC)PtCl_4_(amine) complexes were observed at δ_Pt_ −900 to −800 ppm. All other NHC–Pt(II) complexes displayed a chemical shift below −3304 ppm. The ^195^Pt NMR spectroscopy could then be used to monitor the kinetics and the mechanism of such platinum complexes with biological substances.

## Figures and Tables

**Table 1:** Half-inhibitory concentrations IC_50_ (µM) of the selected complexes toward the HCT116, MCF7 and PC3 cancer cells.

Complex Number	Structure	IC_50_ (µM) HCT116 ^1^	IC_50_ (µM) MCF7 ^1^	IC_50_ (µM) PC3 ^1^
**Cisplatin**	(NH_3_)_2_PtCl_2_	3.57 ± 0.1	4.15 ± 0.7	3.10 ± 0.2
**2**	(NHC)PtBr_2_(pyr)	5.44 ± 1	7.73 ± 1	5.35 ± 1.6
**3**	(NHC)PtBr_2_(DMSO)	>100	>100	>100
**4**	(NHC)PtCl_2_(DMSO)	63 ± 5	80 ± 13	65 ± 6
**5**	(NHC)PtCl_2_(pyr)	3.78 ± 0.1	3.48 ± 1	4.40 ± 0.9
**6**	(NHC)PtBr_4_(amine)	7.5 ± 0.3	23 ± 5	10 ± 1
**8**		14 ± 2	5 ± 1	5 ± 1
**12**		11 ± 0.3	3 ± 0.7	2 ± 0.5
**16**		81.09 ± 2	17.22 ± 1.8	5.42 ± 0.5
**19**		5 ± 1	4 ± 0.2	5 ± 1
**22**	(NHC)PtCl_4_(amine)	0.5 ± 0.03	0.5 ± 0.09	1 ± 0.1
**25**		1.48 ± 0.2	1.78 ± 0.6	1.31 ± 0.2

^1^ HCT116, colon cancer cells; MCF7, breast carcinoma; PC3, prostate adenocarcinoma. (After 72 h of incubation; stock solutions in DMSO for all complexes; stock solution in H_2_O for cisplatin).

**Table 2:** Chemical shift evolution of the Pt signal as a function of the metal oxidation state, the coordination sphere of the metal and the NHC substituents (external reference for ^195^Pt: H_2_PtCl_6_ in D_2_O: δ_Pt_ = 0 ppm).

Complex	Ox. State	δ_Pt_ (ppm) ^195^Pt NMR	δ_C_ (ppm) ^13^C NMR
**1**	+II	−4313	125.1
**2**	+II	−3814	138.2
**3**	+II	−3356	154.7
**4**	+II	−3351	n.o. **^1^**
**5**	+II	−3304	n.o.
**6**	+IV	−2196	n.o.
**7**	+IV	−2168	113.4
**8**	+IV	−2168	115.2
**9**	+IV	−2167	133.9
**10**	+IV	−2083	124.6
**11**	+IV	−2081	n.o.
**12**	+IV	−2080	112.7
**13**	+IV	−2079	115.4
**14**	+IV	−2070	n.o.
**15**	+IV	−2067	n.o.
**16**	+IV	−2063	110.8
**17**	+IV	−2058	110.7
**18**	+IV	−2048	109.3
**19**	+IV	−2040	109.2
**20**	+IV	−2032	n.o.
**21**	+IV	−1901	n.o.
**22**	+IV	−883	n.o.
**23**	+IV	−853	n.o.
**24**	+IV	−825	112.9
**25**	+IV	−810	111.5
**26**	+IV	−795	n.o.

^1^ n.o.: not observed.

## Supplementary Materials

The following are available online. ^195^Pt NMR spectra and characterization for all compounds.

## References

[1] Gietema J.A., Meinardi M.T., Messerschmidt J., Gelevert T., Alt F., Uges D., Seijfer D.T. (2000). Circulating plasma platinum more than 10 years after cisplatin treatment for testicular cancer. Lancet.

[2] Cheff D.M., Hall M.D. (2017). A Drug of Such Damned Nature. 1 Challenges and Opportunities in Translational Platinum Drug Research: Miniperspective. J. Med. Chem..

[3] Um I.S., Armstrong-Gordon E., Moussa Y.E., Gnjidic D., Wheate N.J. (2019). Platinum drugs in the Australian cancer chemotherapy healthcare setting: Is it worthwhile for chemists to continue to develop platinums?. Inorg. Chim. Acta.

[4] Gibson D. (2019). Multi-action Pt (IV) anticancer agents; do we understand how they work?. J. Inorg. Biochem..

[5] Hall M.D., Hambley T.W. (2002). Platinum (IV) antitumour compounds: Their bioinorganic chemistry. Coord. Chem. Rev..

[6] Bouché M., Bonnefont A., Achard T., Bellemin-Laponnaz S. (2018). Exploring diversity in platinum (IV) N-heterocyclic carbene complexes: Synthesis, characterization, reactivity and biological evaluation. Dalton Trans..

[7] Bouché M., Dahm G., Wantz M., Fournel S., Achard T., Bellemin-Laponnaz S. (2016). Platinum (IV) N-heterocyclic carbene complexes: Their synthesis, characterisation and cytotoxic activity. Dalton Trans..

[8] Chardon E., Dahm G., Guichard G., Bellemin-Laponnaz S. (2012). Derivatization of preformed platinum N-heterocyclic carbene complexes with amino acid and peptide ligands and cytotoxic activities toward human cancer cells. Organometallics.

[9] Bellemin-Laponnaz S. (2020). N-Heterocyclic Carbene Platinum Complexes: A Big Step Forward for Effective Antitumor Compounds. Eur. J. Inorg. Chem..

[10] Priqueler J.R.L., Butler I.S., Rochon D.D. (2006). High selectivity of colorimetric detection of p-nitrophenol based on Ag nanoclusters. Appl. Spectrosc. Rev..

[11] Höfer D., Varbaniv H.P., Hejl M., Jakupec M.A., Roller A., Galanski M., Keppler B.K. (2017). Impact of the equatorial coordination sphere on the rate of reduction, lipophilicity and cytotoxic activity of platinum (IV) complexes. J. Inorg. Biochem..

[12] Johnstone T.C., Suntharalingam K., Lippard S.J. (2016). The next generation of platinum drugs: Targeted Pt (II) agents, nanoparticle delivery, and Pt (IV) prodrugs. Chem. Rev..

[13] Johnstone T.C., Alexander S.M., Wilson J.J., Lippard S.J. (2015). Oxidative halogenation of cisplatin and carboplatin: Synthesis, spectroscopy, and crystal and molecular structures of Pt (IV) prodrugs. Dalton Trans..

[14] Bokach N.A., Kukushkin V.Y., Kuznetsov M.L., Garnovskii D.A., Natile G., Pombeiro A.J.L. (2002). Direct addition of alcohols to organonitriles activated by ligation to a platinum (IV) center. Inorg. Chem..

[15] Still B.M., Anil Kumar P.G., Aldrich-Wright J.R., Price W.S. (2007). 195Pt NMR—Theory and application. Chem. Soc. Rev..

[16] Hu D., Yang C., Lok C.-N., Xing F., Lee P.-Y., Fung Y.M.E., Jiang H., Che C.-M. (2019). An Antitumor Bis (N-Heterocyclic Carbene) Platinum (II) Complex That Engages Asparagine Synthetase as an Anticancer Target. Angew. Chem. Int. Ed..

[17] Matczuk M., Ruzik L., Alekssanko S.S., Keppler B.K., Jarosz M., Timerbaev A.R. (2019). Analytical methodology for studying cellular uptake, processing and localization of gold nanoparticles. Anal. Chim. Acta.

[18] Galvez L., Theiner S., Grabarics M., Kowol C.R., Keppler B.K., Hann S., Koellensperger G. (2018). Critical assessment of different methods for quantitative measurement of metallodrug-protein associations. Anal. Bioanal. Chem..

[19] Ahmad S. (2017). Kinetic aspects of platinum anticancer agents. Polyhedron.

[20] Hall M.D., Daly H.L., Zhang J.Z., Zhang M., Alderden R.A., Pursche D., Foran G.J., Hambley T.W. (2012). Quantitative measurement of the reduction of platinum (IV) complexes using X-ray absorption near-edge spectroscopy (XANES). Metallomics.

[21] Czapla-Masztafiak J., Kubas A., Kayser Y., Fernandes D.L.A., Kwiatek W.M., Lipiec E., Deacon G.B., Al-Jorani K., Wood B.R., Szlachetko J. (2018). Mechanism of hydrolysis of a platinum (IV) complex discovered by atomic telemetry. J. Inorg. Biochem..

[22] Huynh H.V. (2017). The Organometallic Chemistry of N-heterocyclic Carbenes.

[23] Teng Q.Q., Huynh H.V. (2017). A Unified Ligand Electronic Parameter Based on 13 C NMR Spectroscopy of N-heterocyclic Carbene Complexes. Dalton Trans..

[24] Teng Q.Q., Ng P.S., Leung J.N., Huynh H.V. (2019). Donor strengths determination of pnictogen and chalcogen ligands by the Huynh electronic parameter and its correlation to sigma Hammett constants. Chem. Eur. J..

[25] Tsipis A.C., Karapetsas I.N. (2017). Prediction of 195Pt NMR of photoactivable diazido- and azine-Pt(IV) anticancer agents by DFT computational protocols. Magn. Reson. Chem..

[26] Appleton T.G., Berry R.D., Davis C.A., Hall J.R., Kimlin H.A. (1984). Reactions of platinum(II) aqua complexes. I: Multinuclear (195Pt, 15N, and 31P) NMR study of reactions between the cis-diamminediaquaplatinum(II) cation and the oxygen-donor ligands hydroxide, perchlorate, nitrate, sulfate, phosphate, and acetate. Inorg. Chem..

[27] Appleton T.G., Hall J.R., Ralph S.F., Thompson C.S.M. (1984). Reactions of platinum(II) aqua complexes. 2. Platinum-195 NMR study of reactions between the tetraaquaplatinum(II) cation and chloride, hydroxide, perchlorate, nitrate, sulfate, phosphate, and acetate. Inorg. Chem..

[28] Benhamou L., Chardon E., Lavigne G., Bellemin-Laponnaz S., César V. (2009). Synthetic routes to N-heterocyclic carbene precursors. Chem. Rev..

[29] Sutter K., Autschbach J. (2012). Computational study and molecular orbital analysis of NMR shielding, spin–spin coupling, and electric field gradients of azido platinum complexes. J. Am. Chem. Soc..

[30] Dahm D., Bailly C., Karmazin L., Bellemin-Laponnaz S. (2015). Synthesis, structural characterization and in vitro anti-cancer activity of functionalized N-heterocyclic carbene platinum and palladium complexes. J. Organomet. Chem..

[31] Chardon E., Puleo G.-L., Dahm G., Guichard G., Bellemin-Laponnaz S. (2011). Direct functionalisation of group 10 N-heterocyclic carbene complexes for diversity enhancement. Chem. Commun..

[32] Chtchigrovsky M., Eloy L., Jullien H., Saker L., Ségal-Bendirdjian E., Poupon J., Bombard S., Cresteil T., Retailleau P., Marinetti A. (2013). Antitumor trans-N-Heterocyclic Carbene–Amine–Pt(II) Complexes: Synthesis of Dinuclear Species and Exploratory Investigations of DNA Binding and Cytotoxicity Mechanisms. J. Med. Chem..

[33] Skander M., Retailleau P., Bourri B., Schio L., Mailliet P., Marinetti A. (2010). N-heterocyclic carbene-amine Pt (II) complexes, a new chemical space for the development of platinum-based anticancer drugs. J. Med. Chem..

[34] Becker M., Port R.E., Zabel H.-J., Zeller W.J., Bachert P. (1998). Monitoring Local Disposition Kinetics of Carboplatinin Vivoafter Subcutaneous Injection in Rats by Means of ^195^Pt NMR. J. Magn. Reson..

