# Supplementary material for: N-Heterocyclic Carbene Platinum(IV) as Metallodrug Candidates: Synthesis and 195Pt NMR Chemical Shift Trend
Source: Molecules. 2020 Jul 9;25(14):3148. doi: 10.3390/molecules25143148 (PMC7397185; doi:10.3390/molecules25143148)
Supplement: Supplementary file 1 [file molecules-25-03148-s001.pdf]

Supporting Information

N-Heterocyclic Carbene Platinum(IV) metallodrug candidates: synthesis ^{195}Pt NMR chemical shift trend.

M. Bouché,[1] B. Vincent,[2] T. Achard,[1] S. Bellemin-Laponnaz[1*]

[1] Institut de Physique et Chimie des Matériaux de Strasbourg, Université de Strasbourg-CNRS UMR7504, 23 rue du Loess, BP 43, 67034 Strasbourg Cedex 2, France.

Fax : +33 388107246 ; Tel : +33 388107166

E-mail: bellemin@unistra.fr

[2] Service de RMN, Fédération de Chimie Le Bel, Université de Strasbourg, 1 rue du Blaise Pascal, BP 296R8, 67008 Strasbourg Cedex, France.

Summary :

A) **General remarks**

B) **Synthesis of NHC-Pt(II) complexes**

B.1. Characterization of complex 1

B.2. Characterization of complex 2

B.3. Characterization of complex 3

B.4. Characterization of complex 4

B.5. Characterization of complex 5

C) **Synthesis of (NHC)PtBr$_4$(amine) complexes**

C.1. Characterization of complex 6

C.2. Characterization of complex 7

C.3. Characterization of complex 8

C.4. Characterization of complex 9

C.5. Characterization of complex 10

C.6. Characterization of complex 11

C.7. Characterization of complex 12

C.8. Characterization of complex 13

C.9. Characterization of complex 14

C.10. Characterization of complex 15

C.11. Characterization of complex 16

C.12. Characterization of complex 17

C.13. Characterization of complex 18

C.14. Characterization of complex 19

C.15. Characterization of complex 20

C.16. Characterization of complex 21

D) Synthesis of (NHC)$PtCl_4$(amine) complexes

D.1. Characterization of complex 22

D.2. Characterization of complex 23

D.3. Characterization of complex 24

D.4. Characterization of complex 25

D.5. Characterization of complex 26

E) Molecular structure of complex 15

A) General remarks

All manipulations of air and moisture sensitive compounds were carried out using standard Schlenk techniques under an argon atmosphere and solvents were purified and degassed following standard procedures. All reagents were purchased from commercial chemical suppliers (Acros, Alfa Aesar, and TCI Europe) and used without further purification. ^{1}H and ^{13}C Nuclear Magnetic Resonance (NMR) spectra were recorded on a Brucker AVANCE 300 or Bruker AVANCE 500 spectrometer using the residual solvent peak as reference ($CDCl_3$: $\delta H = 7.26$ ppm; $\delta C = 77.16$ ppm) at 295K. HMQC ^{1}H-^{195}Pt spectra were recorded on a Bruker AVANCE 600 spectrometer using the residual solvent peak as reference for ^{1}H calibration and an external reference for ^{195}Pt (H_2PtCl_6 in D_2O: $\delta Pt = 0$ ppm) at Institut de Chimie NMR Facility of the University of Strasbourg. Positive mode electrospray ionization mass spectra (ESI-HRMS) analyses have been carried out on microTOF, Bruker Daltonics. The purity of the complexes was confirmed by elemental analyses, performed by the 'Service d'analyse élémentaire' of the Strasbourg chemistry department.

B) Synthesis of NHC-platinum complexes

General procedure for the synthesis of [(NHC)PtX$_2$(pyridine)] complexes (X = I, Br or Cl)

The ligand precursor (imidazolium halide, 1.1 equiv.), $PtCl_2$ (1 equiv.), NaI or NaBr or NaCl (10 equiv.) and K_2CO_3 (10 equiv.) were suspended under argon in anhydrous pyridine (10 mL). The mixture was sonicated for 20 min, heated overnight at 100 °C, then concentrated under reduced pressure, dissolved in CH_2Cl_2, and filtered through a Celite plug. The residue was purified by silica gel chromatography (pentane/CH_2Cl_2, 1:1 to CH_2Cl_2) to afford the complexes **1**,**2** and **5** as a yellow powder.

B.1. Characterization of complex 1

1

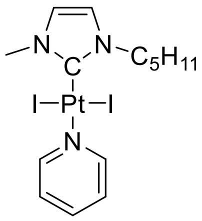

Complex **1** was synthesized according to our reported procedure.[1]

HMQC ^{1}H-^{195}Pt NMR ($CDCl_3$, 64.2 MHz, 20 °C): δ – 4313 (m).

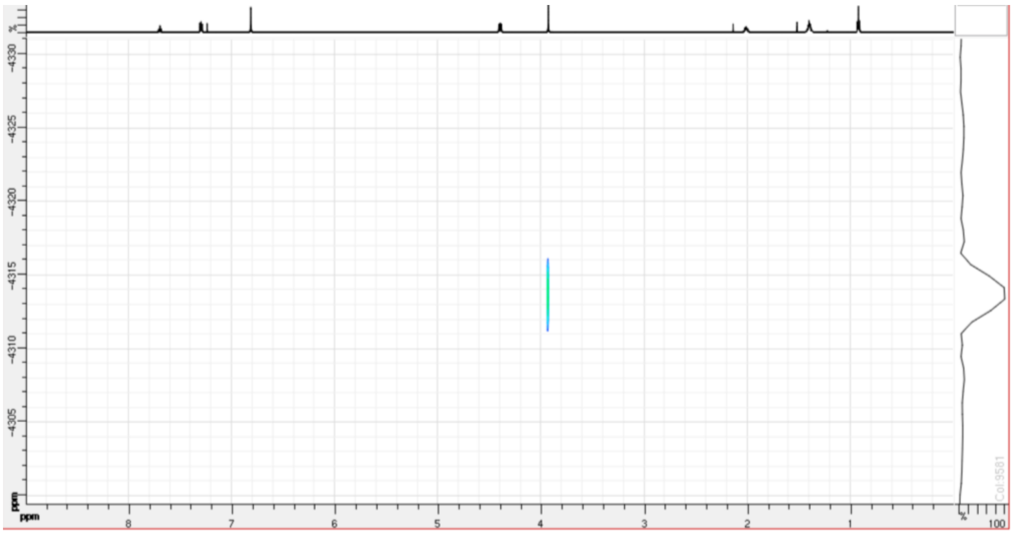

B.2. Characterization of complex 2

2

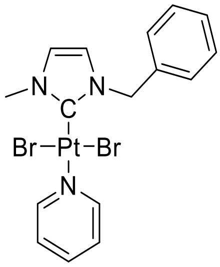

Yellow solid, 59.2 mg, yield 52%. ^{1}H NMR ($CDCl_3$, 300 MHz, 20 °C): δ 4.11 (s, 3H, N-CH_3), 5.83 (s, 2H, N-CH_2), 6.64 (d, J= 2.1Hz, 1H, CH_{im}), 6.83 (d, J=2.1 Hz, 1H, CH_{im}), 7.27-7.53 (m, 7H, 5H_{ar} and H_{pyr}), 7.76 (tt, *J1*=7.6 Hz, *J2*=1.6 Hz, 1H, H_{pyr}), 9.04 (dt, *J1*=5.0 Hz, *J2*=1.6 Hz, 2H, H_{pyr}); ^{13}C NMR ($CDCl_3$, 75 MHz, 20 °C): δ 37.9 (N-CH_3), 54.2 (N-CH_2), 120.0 (CH_{im}), 122.4 (CH_{im}), 124.9 (C_{pyr}), 128.2 (CH_{ar}), 128.8 (CH_{ar}), 128.8 (CH_{ar}), 135.7 (C_{ar}), 137.7 (C_{pyr}), 138.2 (C-Pt), 152.6 (C_{pyr}); HMQC ^{1}H-^{195}Pt NMR ($CDCl_3$, 64.2 MHz, 20 °C): *δ* – 3814 (m).

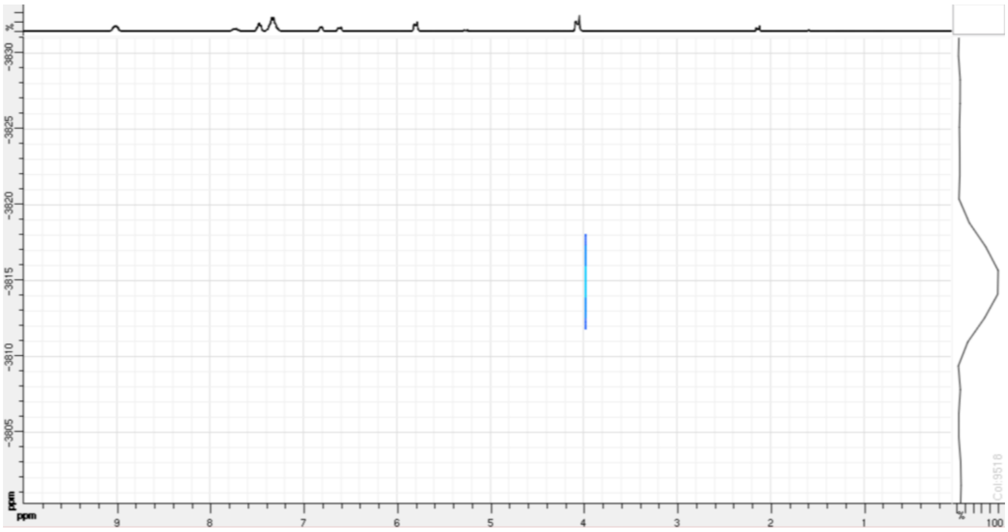

General procedure for the synthesis of cis [(NHC)PtX_2(DMSO)] (X = Br or Cl)

The [(NHC)PtX_2(DMSO)] complexes **3** and **4** were synthesized according to reported procedure.[2] A solution of bis(benzyl)imidazol-2-ylidene silver(I) bromide (20 mg, $4.42x10^{-5}$ mol) in DMSO was treated with K_2PtCl_4 (19.3 mg, $4.64x10^{-5}$ mol) and the resulting mixture was stirred at 60 ℃ for 24 h. After adding CH_2Cl_2 the reaction mixture was filtered, and the filtrate was washed with water and then dried over Na_2SO_4. The solvent was removed in vacuum and the remainder recrystallized from CH_2Cl_2/pentane.

B.3. Characterization of complex 3

3

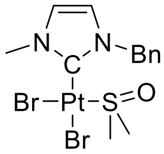

Colourless oil, quant. ^{1}H NMR ($CDCl_3$, 300 MHz, 20 ℃): δ 3.12 (s+d, J=12.6 Hz, 3H, S- CH_3), 3.57 (s+d, J=12.6 Hz, 3H, S- CH_3), 4.00 (s, 3H, N- CH_3), 5.44 (d, J=15.4 Hz, 1H, N-CH_2), 5.81 (d, J=15.4 Hz, 1H, N- CH_2), 6.84 (d, J=1.8 Hz, 1H, CH_{im}), 6.94 (d, J=1.8 Hz, 1H, CH_{im}), 7.34 (m, 5H, H_{ar}); ^{13}C NMR ($CDCl_3$, 75 MHz, 20 ℃): δ 37.9 (N- CH_3), 46.0 (S- CH_3), 47.0 (S-

CH_3), 54.3 (N- CH_2), 121.2 (CH_{im}), 122.4 (CH_{im}), 128.1 (C H_{ar}), 128.5 (C H_{ar}), 129.1 (C H_{ar}), 135.2 (Car), 154.7 (C-Pt); HMQC ^1H-^{195}Pt NMR ($CDCl_3$, 64.2 MHz, 20 ℃): δ – 3356 (m).

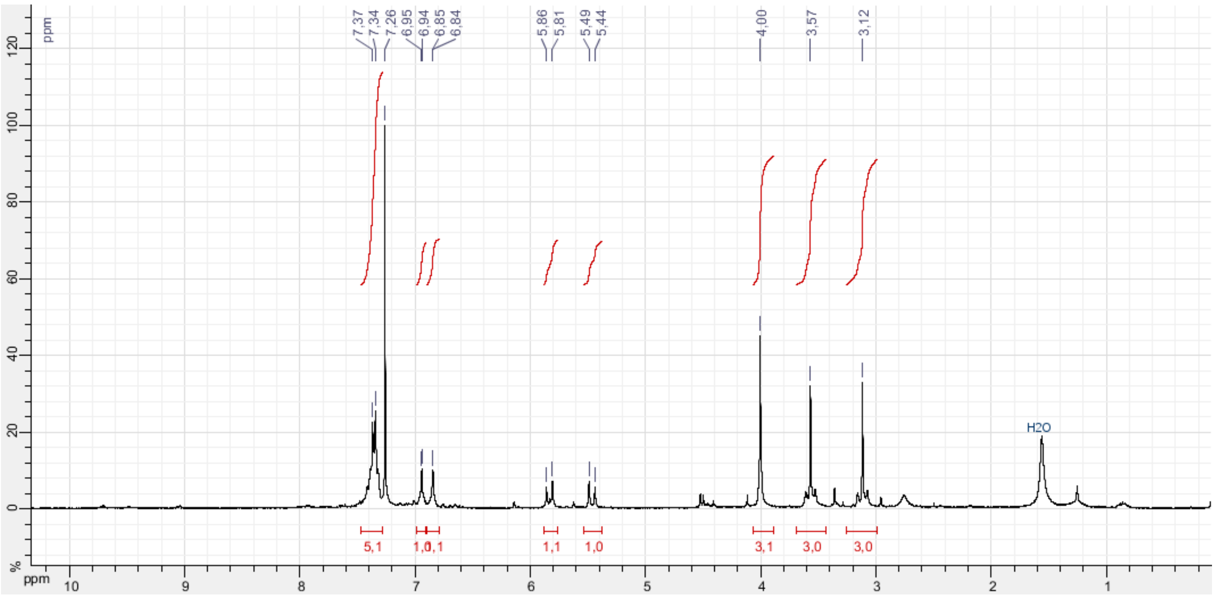

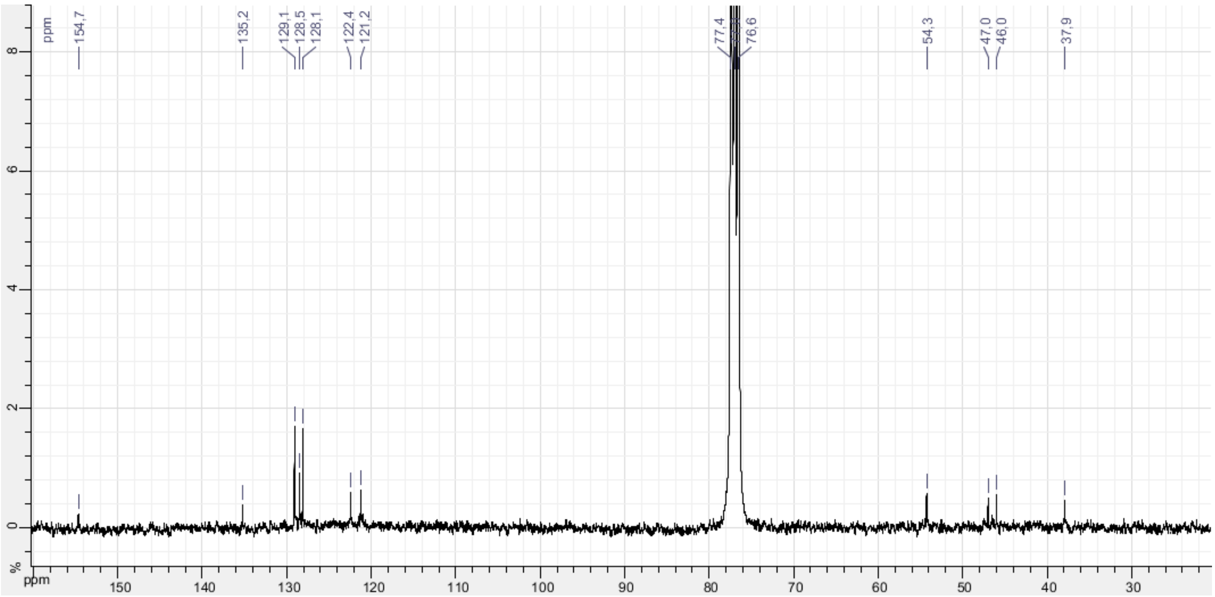

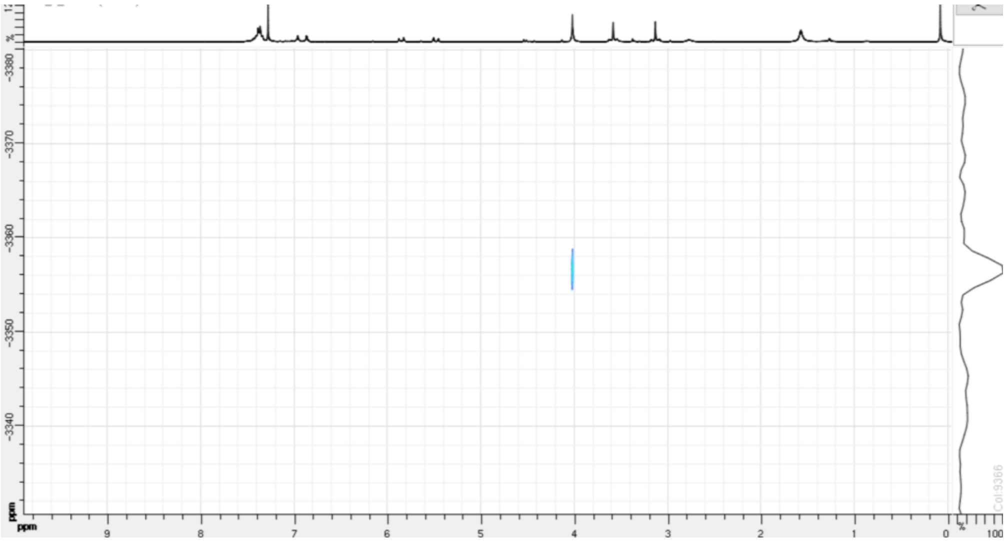

B.4. Characterization of complex 4

4

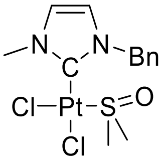

Colourless oil, quant. ^{1}H NMR ($CDCl_3$, 300 MHz, 20 ℃): 3.03 (s+d, J=12.6 Hz, 3H, S- CH_3), 3.48 (s+d, J=12.6 Hz, 3H, S- CH_3), 4.01 (s, 3H, N- CH_3), 5.42 (d, J=15.4 Hz, 1H, N- CH_2), 5.81

(d, J=15.4 Hz, 1H, N- CH_2), 6.86 (d, J=1.8 Hz, 1H, CH_{im}), 6.94 (d, J=1.8 Hz, 1H, CH_{im}), 7.35 (m, 5H, H_{ar}); HMQC ^1H-^{195}Pt NMR ($CDCl_3$, 64.2 MHz, 20 ℃): δ – 3351 (m).

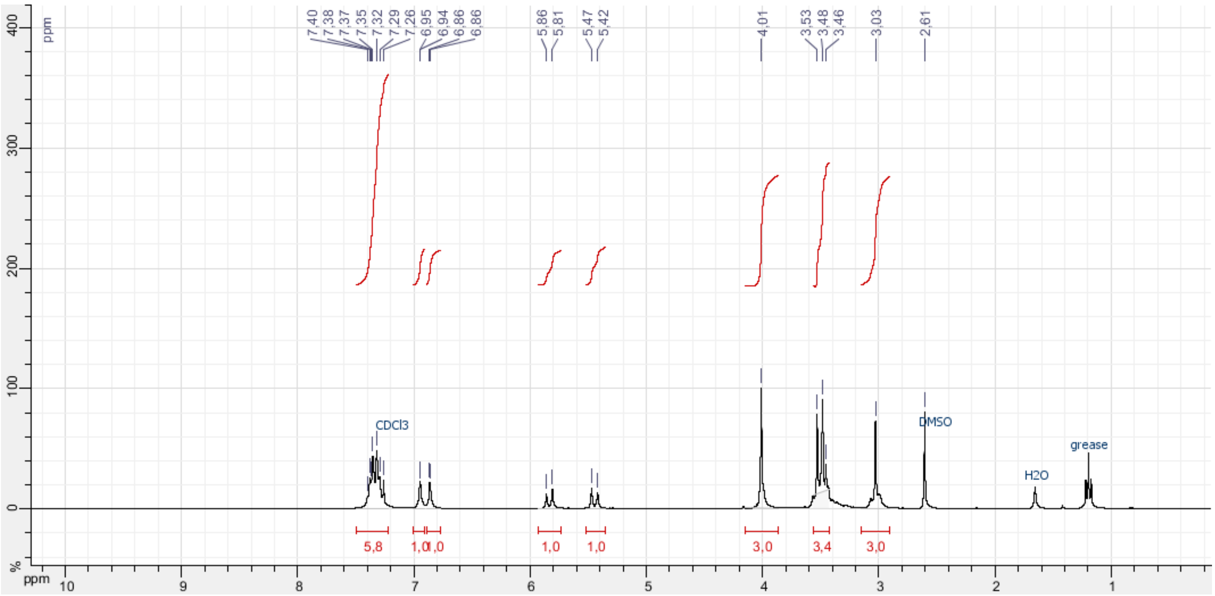

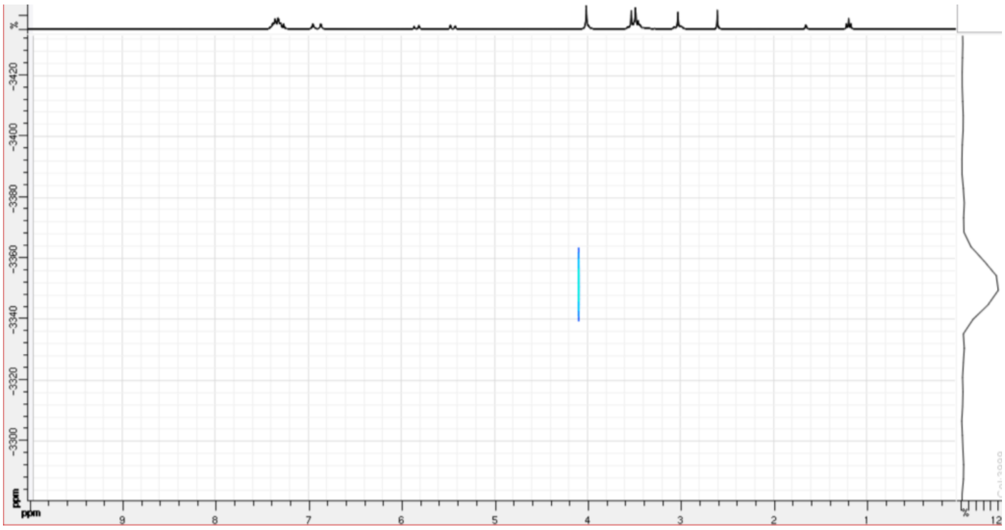

B.5. Characterization of complex 5

5

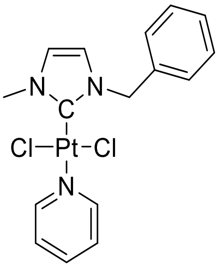

Yellow solid, 23.3 mg, yield 24%. ^{1}H NMR ($CDCl_3$, 300 MHz, 20 ℃): δ 4.09 (s, 3H, N- CH_3), 5.82 (s, 2H, N- CH_2), 6.63 (d, *J*=2.5 Hz, 1H, CH_{im}), 6.82 (d, *J*=2.5 Hz, 1H, CH_{im}), 7.31-7.39 (m, 5H, C H_{ar}), 7.46-7.50 (m, 2H, C H_{pyr}), 7.75 (m, 1H, C H_{pyr}), 9.03 (m, 2H, C H_{pyr}); HMQC ^{1}H-^{195}Pt NMR ($CDCl_3$, 64.2 MHz, 20 ℃): δ – 3304 (m).

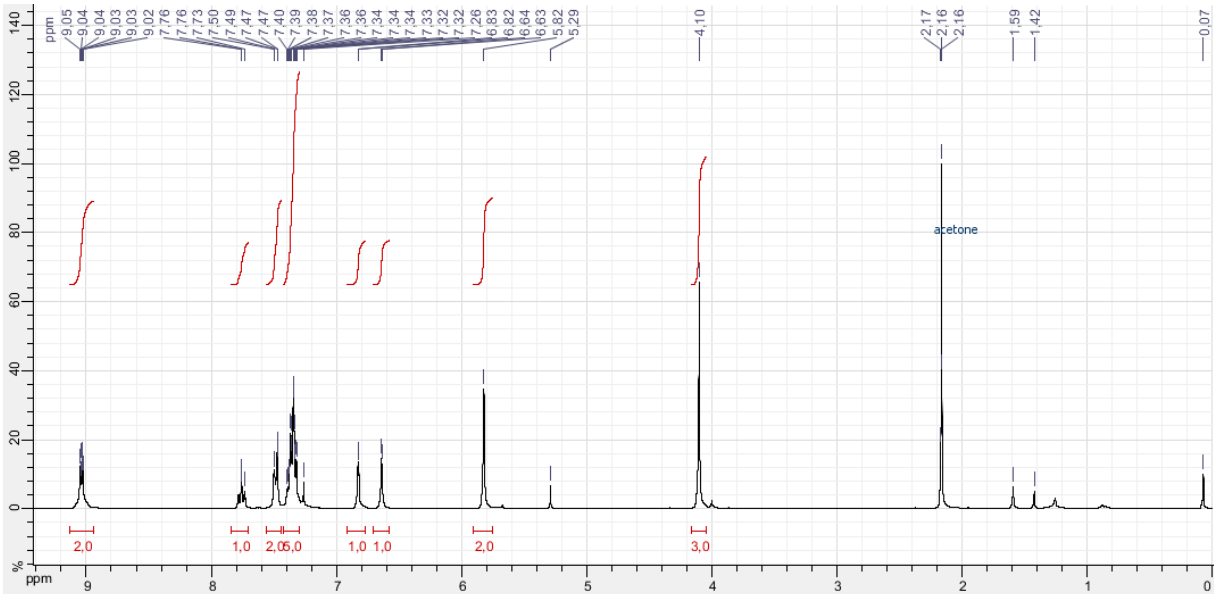

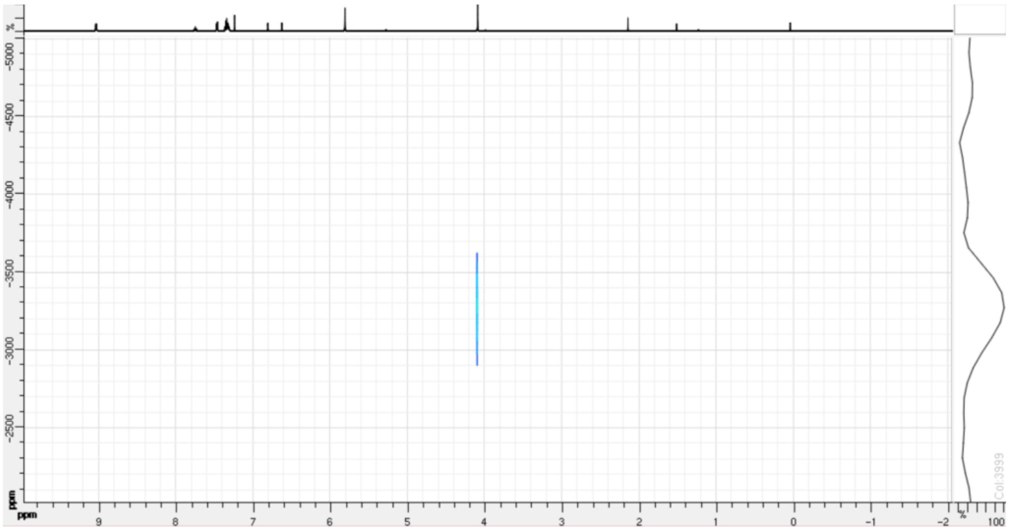

C) **Synthesis of (NHC)PtBr4(amine) complexes**

General procedure for the synthesis of (NHC)PtBr4(amine) complexes

In a 10 mL round bottom flask, the precursor [(NHC)PtI_2L] (10 mg, 1 equiv.) was dissolved in CH_2Cl_2 (5 mL), cooled at 0 °C and Br_2 (2 equiv.) was slowly added under nitrogen. After 30 min, pentane (10 mL) was added and the resulting red precipitate (**5**-**21**) was filtered off, washed and dried.

C.1. Characterization of complex 6

6

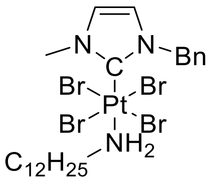

Complex **6** was synthesized according to our reported procedure.[1]

HMQC ^1H-^{195}Pt NMR ($CDCl_3$, 64.2 MHz, 20 °C): δ – 2196 (m).

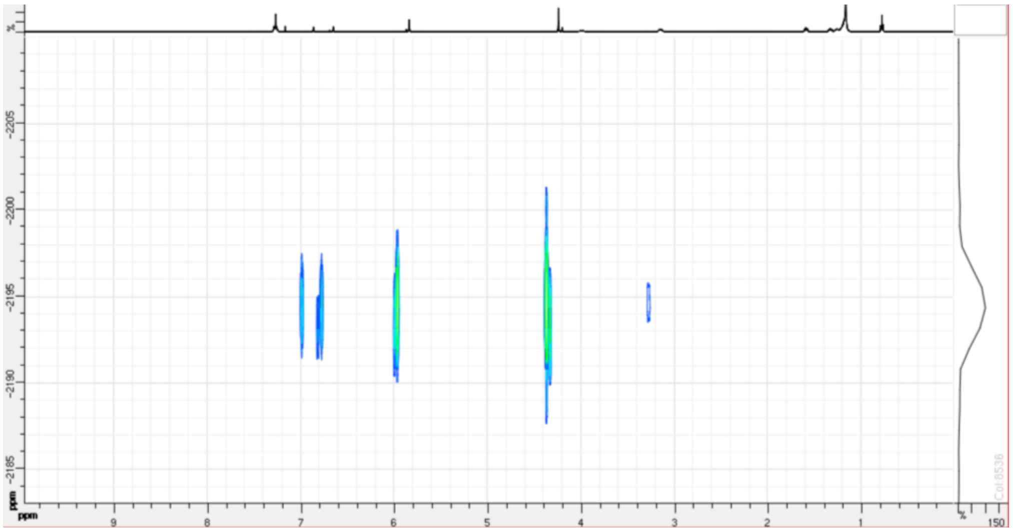

C.2. Characterization of complex 7

7

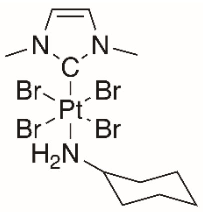

Red solid, 11.2 mg, yield 99%. 1H NMR ($CDCl_3$, 300 MHz, 20 ℃): δ 1.19-1.46 (m, 5H, CH_2), 1.65-1.78 (m, 3H, CH_2), 2.30 (m, 2H, CH_2), 3.55 (bs, 1H, C*H*-NH2), 3.99 (bs, 2H, NH2), 4.32 (s, 6H, N- CH_3), 6.99 (s, 2H, CH_{im}); 13C NMR ($CDCl_3$, 125 MHz, 20 °C): δ 23.6 (CH_2), 24.4 (CH_2), 34.6 (CH_2), 42.9 (s+d, *J*=144.1 Hz, N- CH_3), 53.8 (CH) , 113.4 (s+d, *J*=1036.4 Hz, C-Pt), 124.6 (s+d, *J*=22.5 Hz, CH_{im}) ; HMQC ^{1}H-^{195}Pt NMR ($CDCl_3$, 64.2 MHz, 20 ℃): δ – 2168 (m).

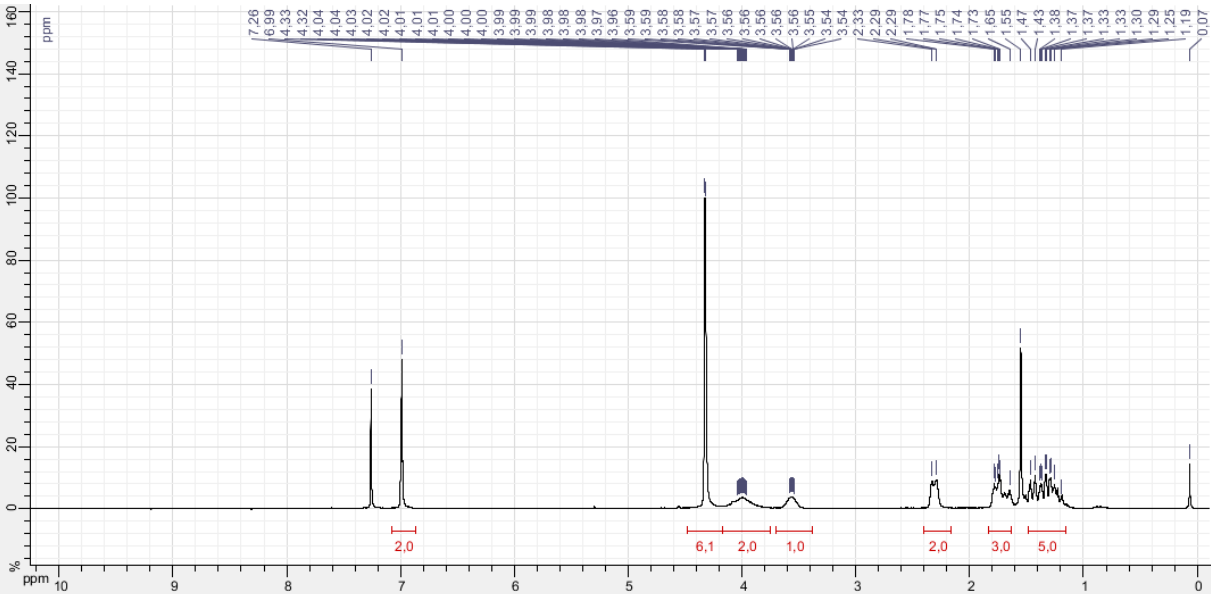

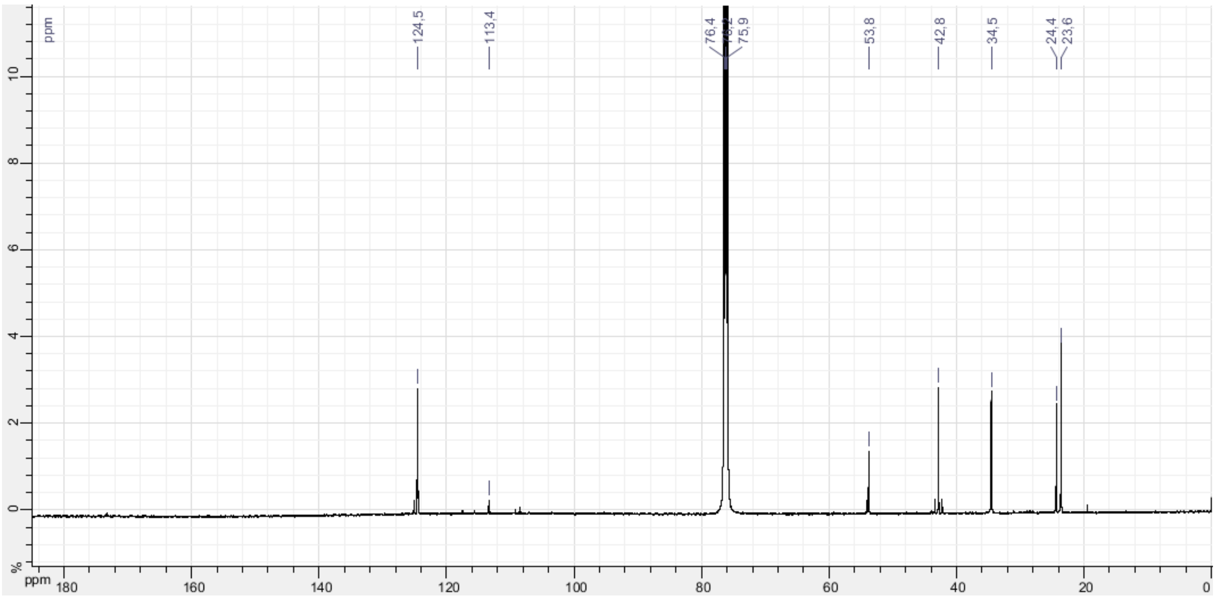

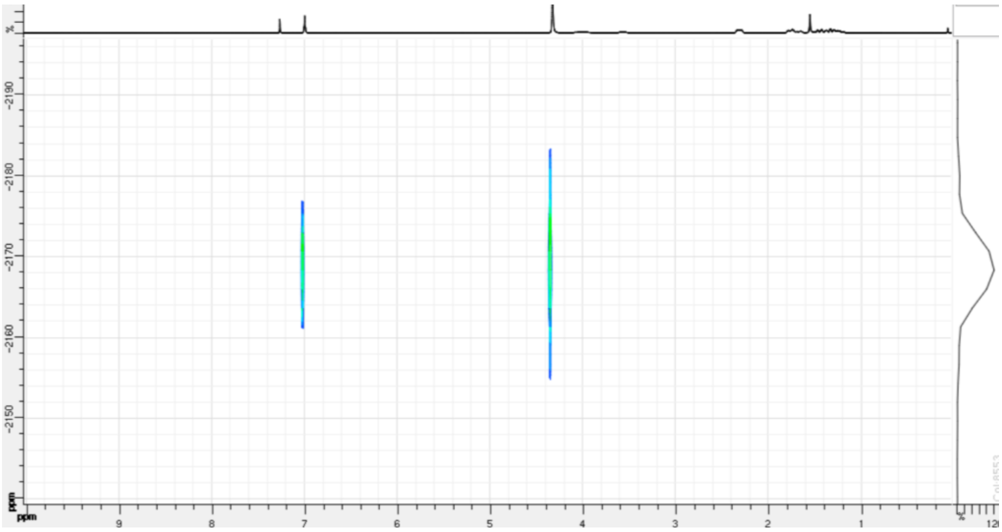

C.3. Characterization of complex 8

8

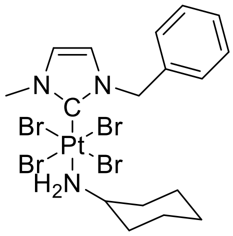

Complex **8** was synthesized according to our reported procedure.[1]

HMQC ^{1}H-^{195}Pt NMR ($CDCl_3$, 64.2 MHz, 20 ℃): δ – 2168 (m).

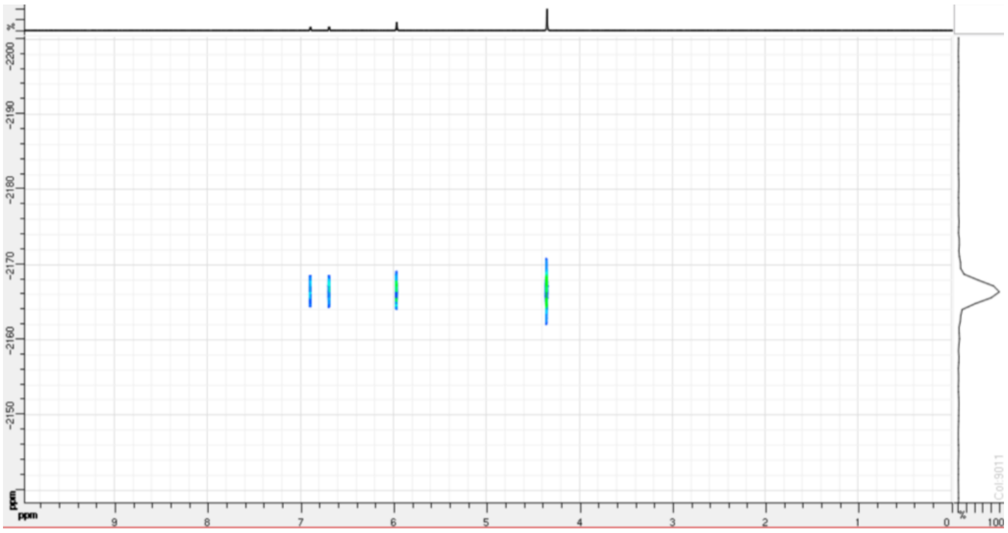

C.4. Characterization of complex 9

9

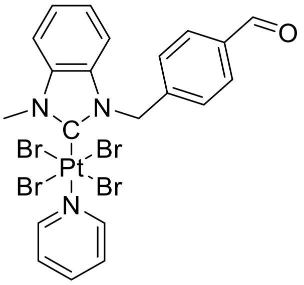

Red solid, 12.1 mg, yield 99%. ^{1}H NMR ($CDCl_3$, 300 MHz, 20 °C): δ 4.73 (s, 3H, N- CH_3), 6.63 (s, 2H, N- CH_2), 6.94 (d, *J*=8.3 Hz, 1H, H_{ar}), 7.18 (t, *J*=7.8 Hz, 1H, H_{ar}), 7.34 (m, 3H, H_{ar}), 7.46 (t, *J*=7.1 Hz, 2H, H_{pyr}), 7.57 (d, *J*=8.3 Hz, 1H, H_{ar}), 7.80 (d, *J*=8.3 Hz, 2H, H_{ar}), 7.89-7.94 (tt, *J*=7.6 Hz, 1H, H_{pyr}), 9.66-9.75 (q, *J*=16.5 Hz et *J*=10.7 Hz, 2H, H_{pyr}), 9.97 (s, 1H, CHO); ^{13}C NMR ($CDCl_3$, 75 MHz, 20°C): δ 41.3 (N- CH_3), 57.2 (N- CH_2), 111.9 (N- C_{im}), 113.2 (N- C_{im}), 124.7 (t, *J*=9.8 Hz),125.1, 125.3, 127.4, 129.8, 133.9 (C-Pt), 135.6, 139.5, 143.1, 154.5, 191.5 (CHO) ; HMQC ^{1}H-^{195}Pt NMR ($CDCl_3$, 64.2 MHz, 20 ℃): *δ* –2167 (m).

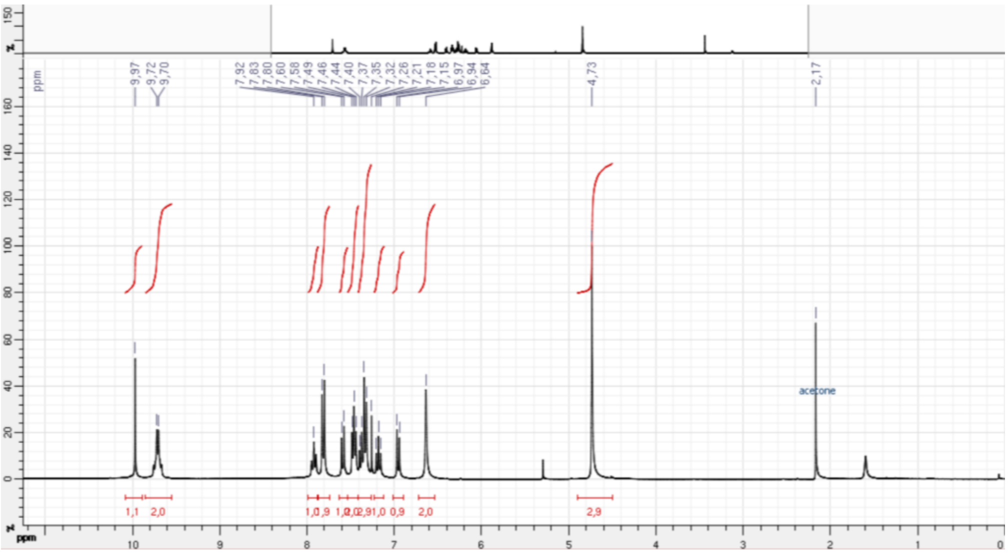

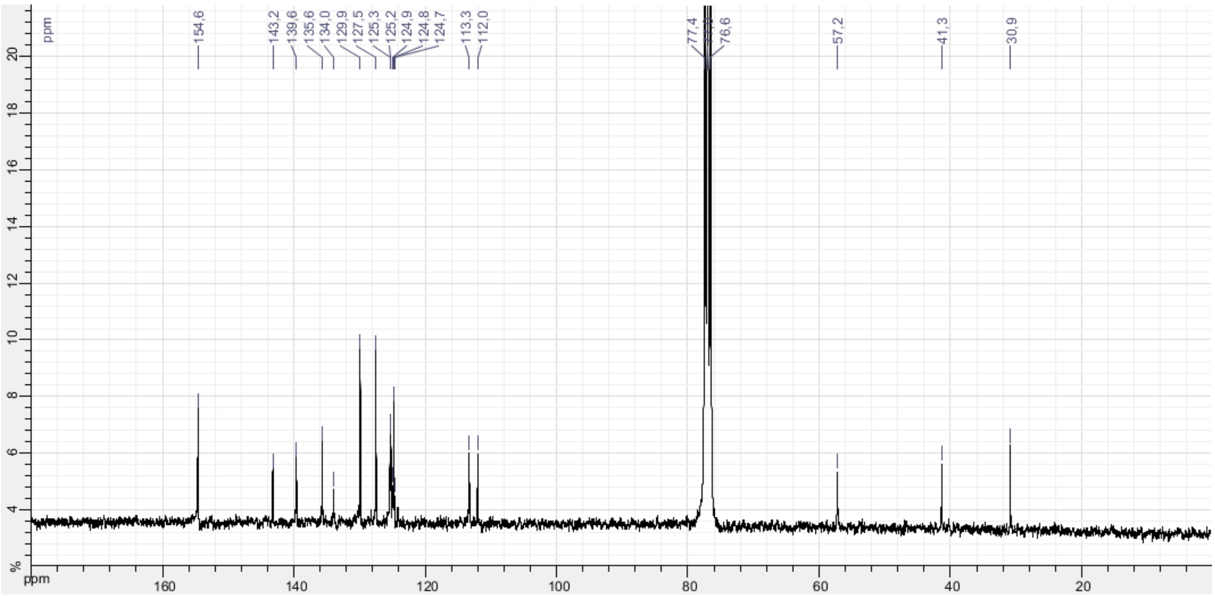

C.5. Characterization of complex 10

10

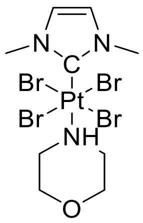

Red solid, 11.0 mg, yield 97%. ^{1}H NMR ($CDCl_3$, 300 MHz, 20 °C): δ 3.42-3.47 (m, 2H, CH_2), 3.65-3.77 (m, 4H, CH_2), 3.87 (bs, 1H, NH), 4.01-4.06 (m, 2H, CH_2), 4.32 (s, 6H, N-

CH_3), 7.01 (s, 2H, CH_{im}); ^{13}C NMR ($CDCl_3$, 75 MHz, 20 ℃): δ 43.0 (N– CH_3), 50.8 (N–CH_2), 69.2 (t, J=19.3 Hz, HN– CH_2), 111.0 (t, J=526.6 Hz, C–Pt), 124.6 (t, J=11.4 Hz, CH_{im}); HMQC $^{1}H–^{195}Pt$ NMR ($CDCl_3$, 64.2 MHz, 20 ℃): δ -2083 (m).

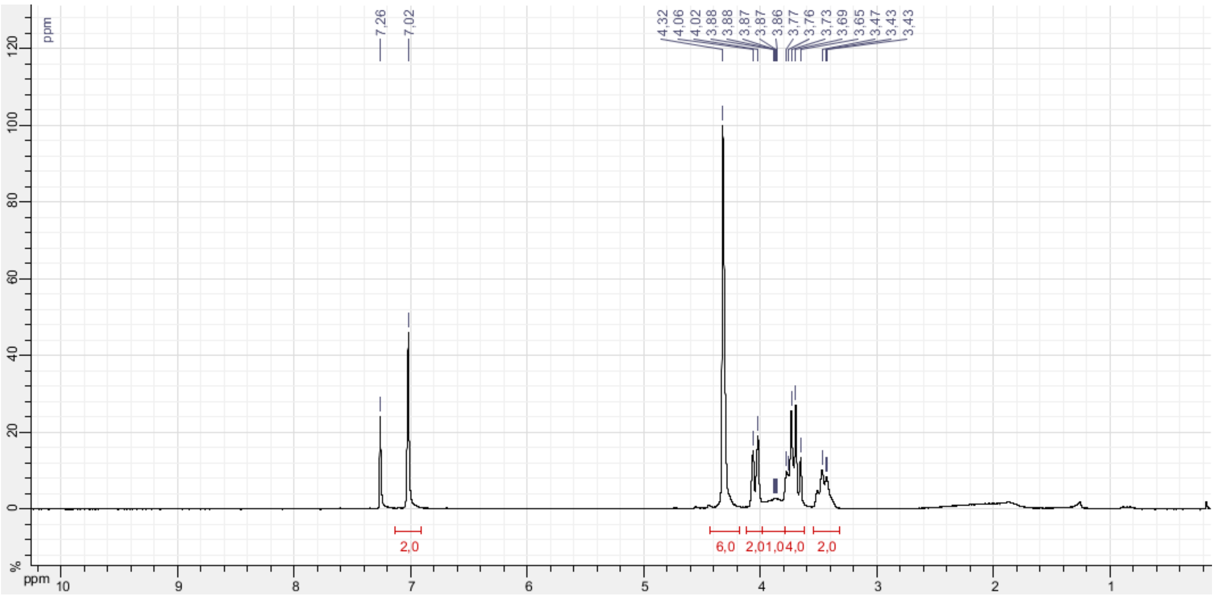

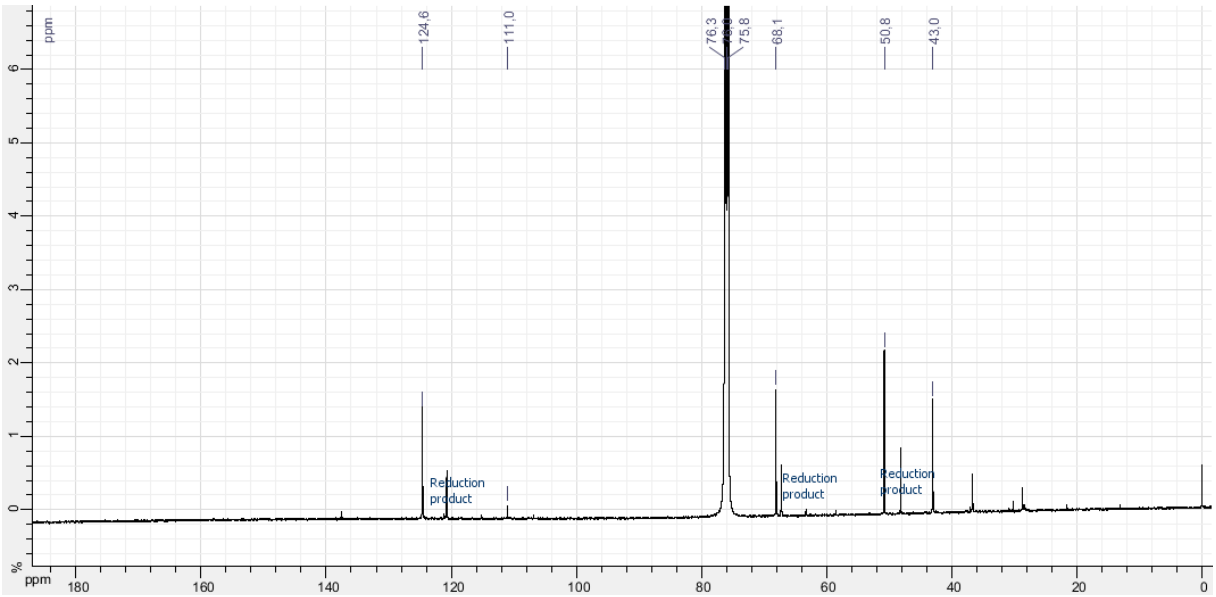

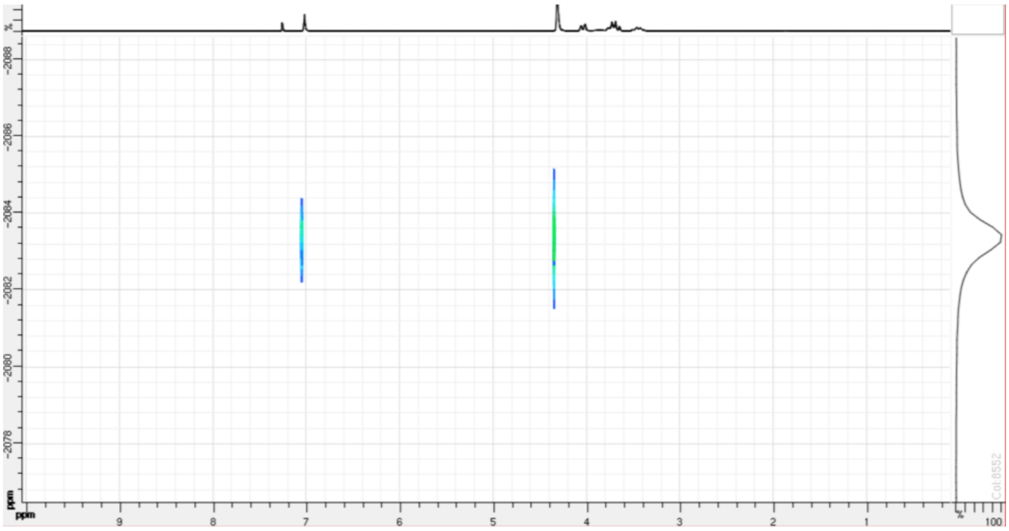

C.6. Characterization of complex 11

11

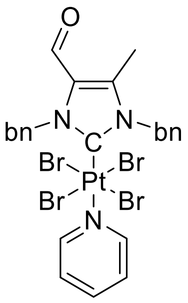

Red solid, 10.9 mg, yield 99%. ^{1}H NMR ($CDCl_3$, 300 MHz, 20 °C): δ 2.24 (s, 3H, CH_3), 6.37 (s, 2H, N- CH_2), 6.64 (s, 2H, N- CH_2), 7.11-7.18 (m, 4H, C H_{ar}), 7.28-7.46 (m, 8H, C H_{pyr} +C H_{ar}), 7.90 (m, 1H, C H_{pyr}), 9.57 (s, 1H, CHO), 9.66 (m, 2H, C H_{pyr}); No ^{13}C NMR could be recorded due to low solubility; HMQC ^{1}H–^{195}Pt NMR ($CDCl_3$, 64.2 MHz, 20 °C): δ −2081 (m).

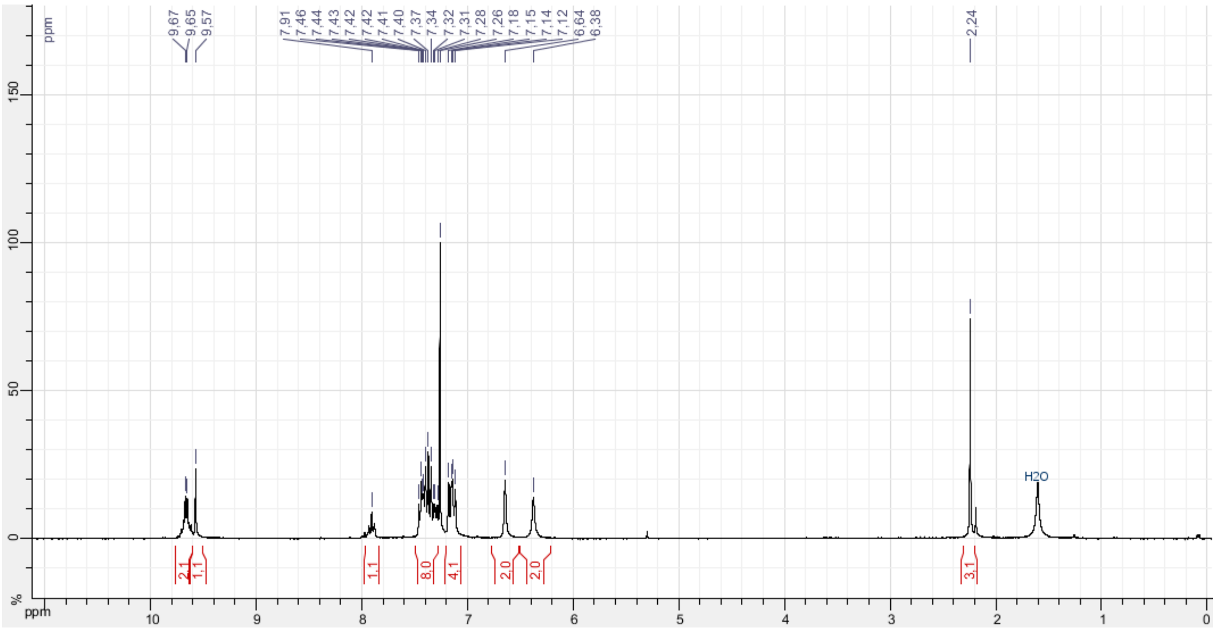

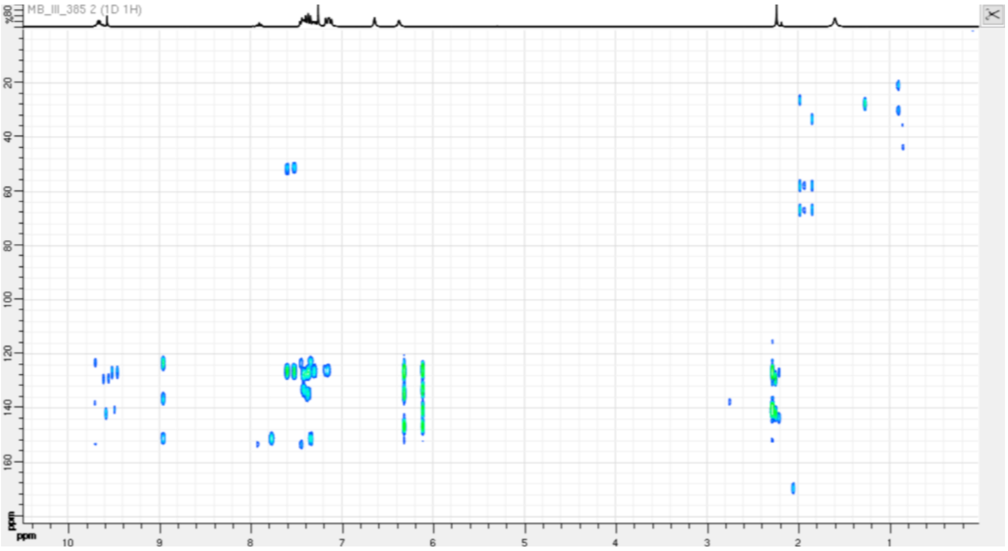

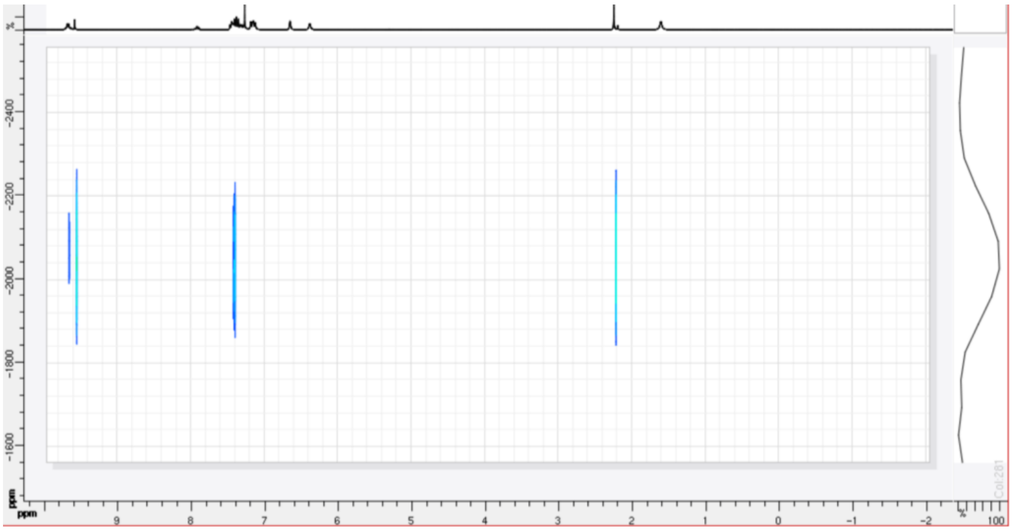

C.7. Characterization of complex 12

12

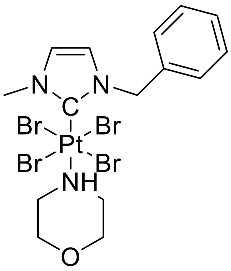

Complex **12** was synthesized according to our reported procedure.[1]

HMQC ^{1}H-^{195}Pt NMR ($CDCl_3$, 64.2 MHz, 20 °C): δ – 2080 (m).

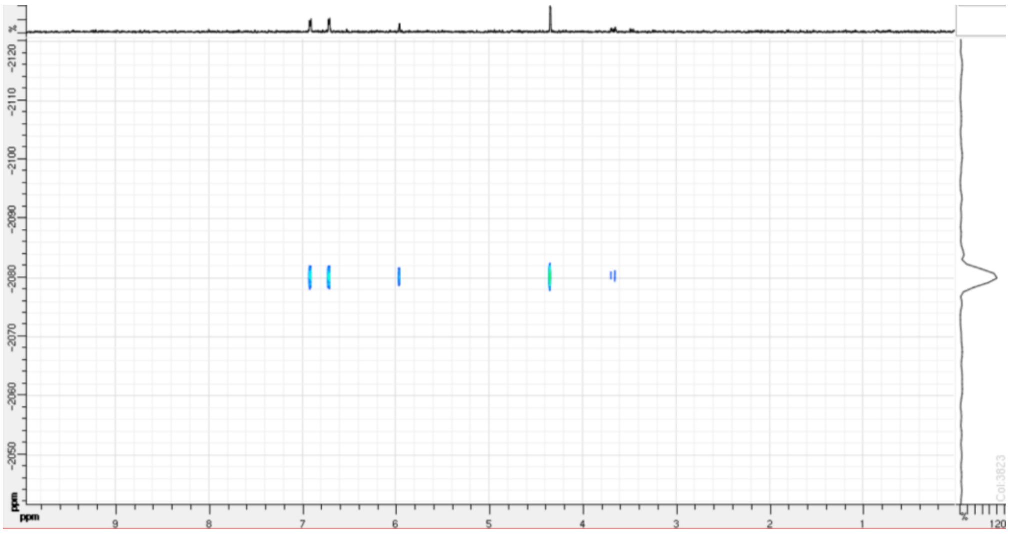

C.8. Characterization of complex 13

13

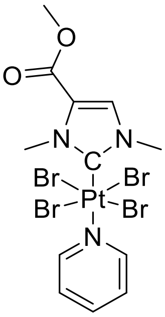

Red solid, 12.5 mg, yield 99%. ^{1}H NMR (CD2Cl2, 300 MHz, 20 ℃): δ 3.91 (s, 3H, O- CH_3), 4.53 (s, 3H, N- CH_3), 4.66 (s, 3H, N- CH_3), 7.47 (t, *J*=7.3 Hz, 2H, C H_{pyr}), 7.72 (t, *J*=3.0 Hz, 1H, CH_{im}), 7.93 (t, *J*=14.6 Hz, 1H, C H_{pyr}), 9.66 (m, 2H, C H_{pyr}); ^{13}C NMR ($CDCl_3$, 75 MHz, 20 ℃): δ 43.1 (N- CH_3), 44.3 (N- CH_3), 51.8 (O- CH_3), 115.4 (t, *J*=1046.8 Hz, C-Pt), 124.2 (s+d, *J*=19.8 Hz, CH_{im}), 125.8 (s+d, *J*=25.0 Hz, C_{im}), 131.3 (s+d, *J*=22.4 Hz, C H_{pyr}), 138.9 (C H_{pyr}), 153.7 (C H_{pyr}), 157.2 (C=O); HMQC ^{1}H-^{195}Pt NMR ($CDCl_3$, 64.2 MHz, 20 ℃): *δ* – 2079 (m).

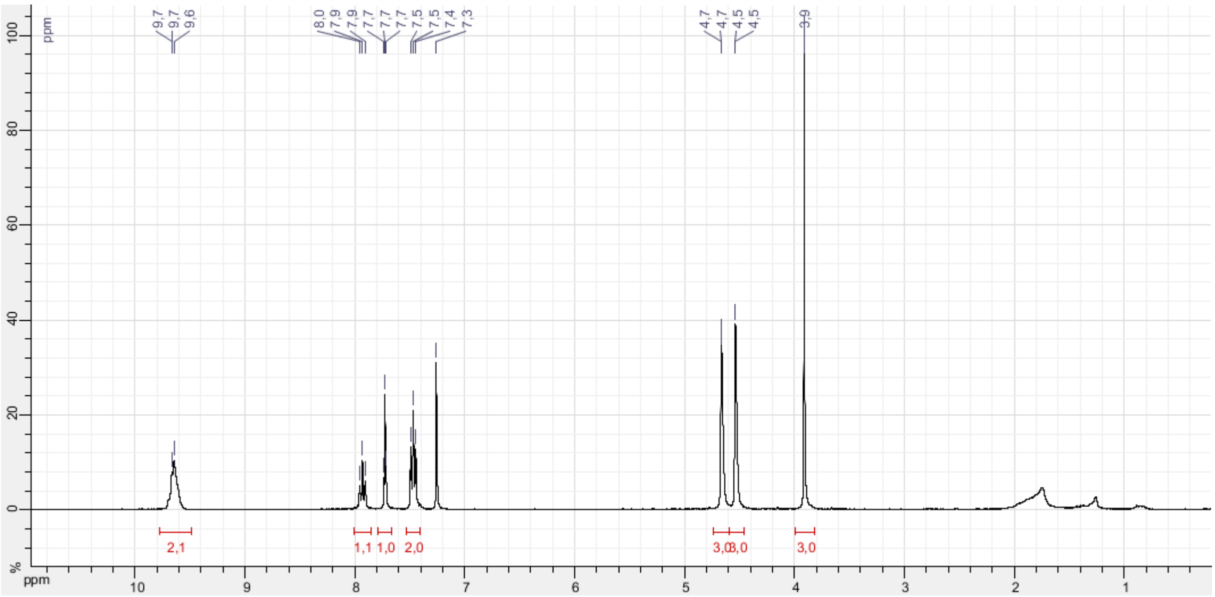
9,7 9,7 9,6
8,0 7,9 7,9 7,7 7,7 7,7 7,5 7,5 7,4 7,3
4,7 4,7 4,5 4,5
3,9
2,1
1,1 1,0 2,0
3,0 3,0
3,0
ppm
%

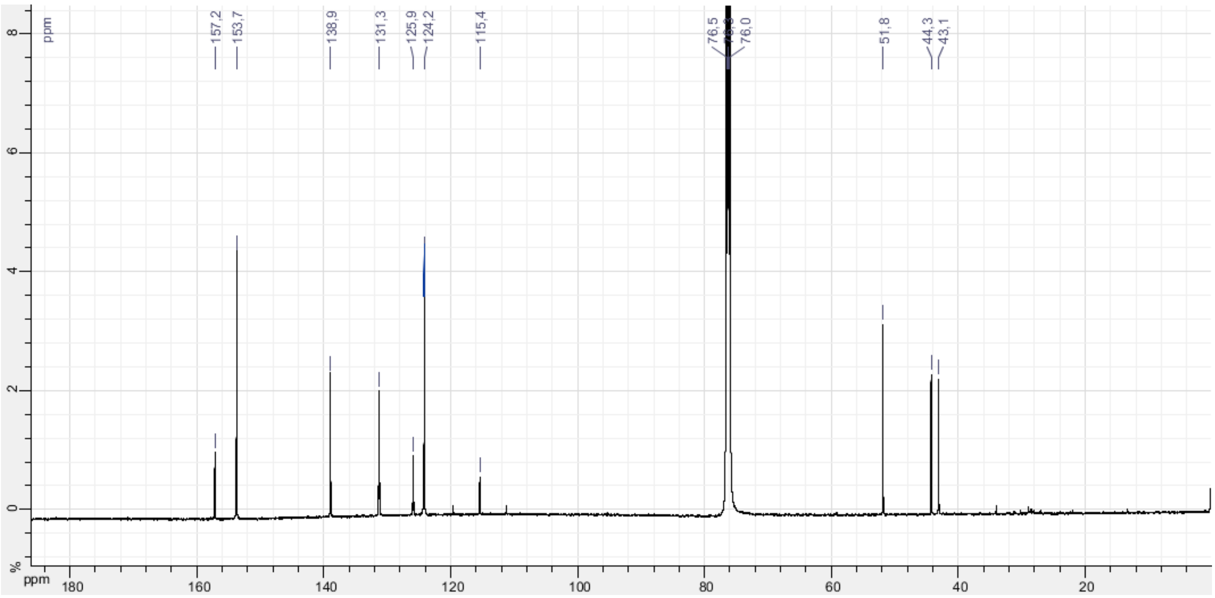
157,2 153,7
138,9
131,3 125,9 124,2
115,4
76,5 76,0
51,8
44,3 43,1
ppm
%

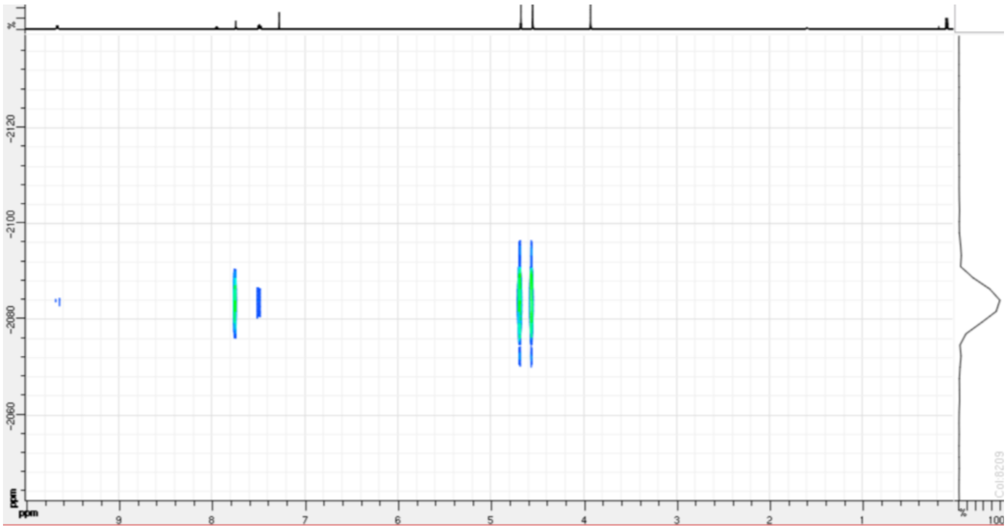
ppm

C.9. Characterization of complex 14

14

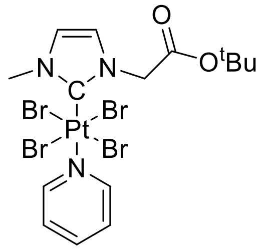

Red solid, 16.7 mg, yield 99%. ^{1}H NMR ($CDCl_3$, 300 MHz, 20 ℃): δ 3.97 (N– CH_3), 5.23 (s, 2H, N– CH_2), 6.87 (d, *J*=2.1 Hz, 1H, CH_{im}), 7.01 (d, *J*=2.1 Hz, 1H, CH_{im}), 7.32 (m, 2H, H_{pyr}), 7.73 (m, 1H, H_{pyr}), 9.03 (m, 2H, H_{pyr}); ^{13}C NMR ($CDCl_3$, 75 MHz, 20 ℃): δ 28.0 (CtBu), 44.1 (N– CH_3), 56.7(N– CH_2), 124.7(s + d, *J*=20.2 Hz, CH_{im}), 126.4 (Cpyr), 139.4 (Cpyr), 154.4 (Cpyr), (C-Pt) and (C=O) not seen; HMQC 1H–195Pt NMR ($CDCl_3$, 64.2 MHz, 20 ℃): δ – 2070 (m).

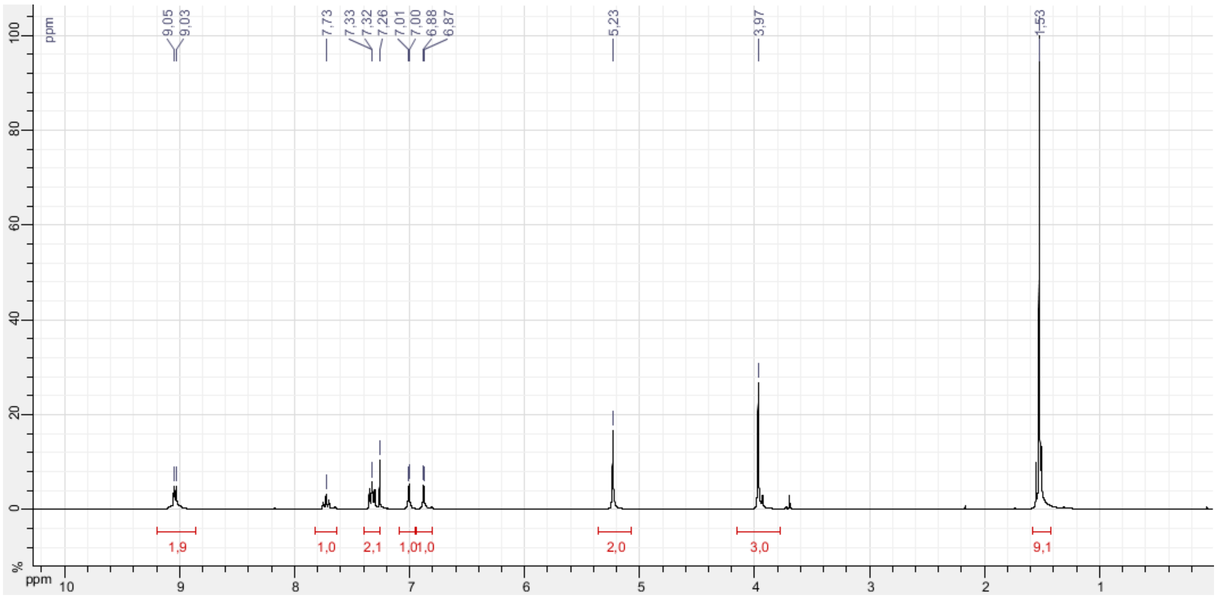

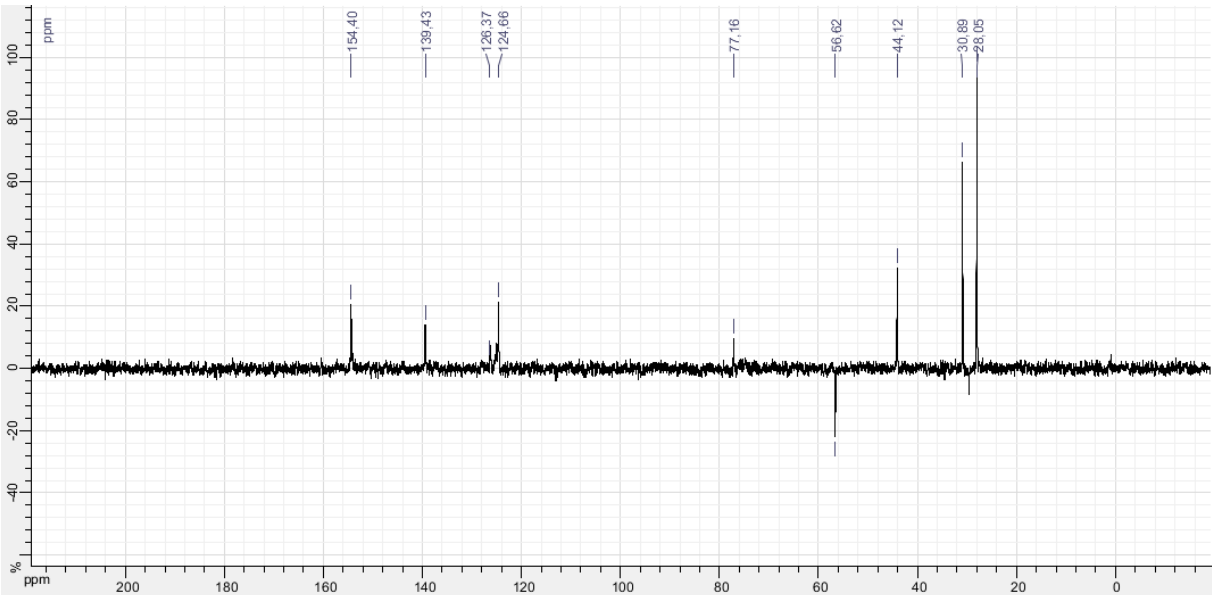
154,40
139,43
126,37
124,66
77,16
56,62
44,12
30,89
28,05
ppm

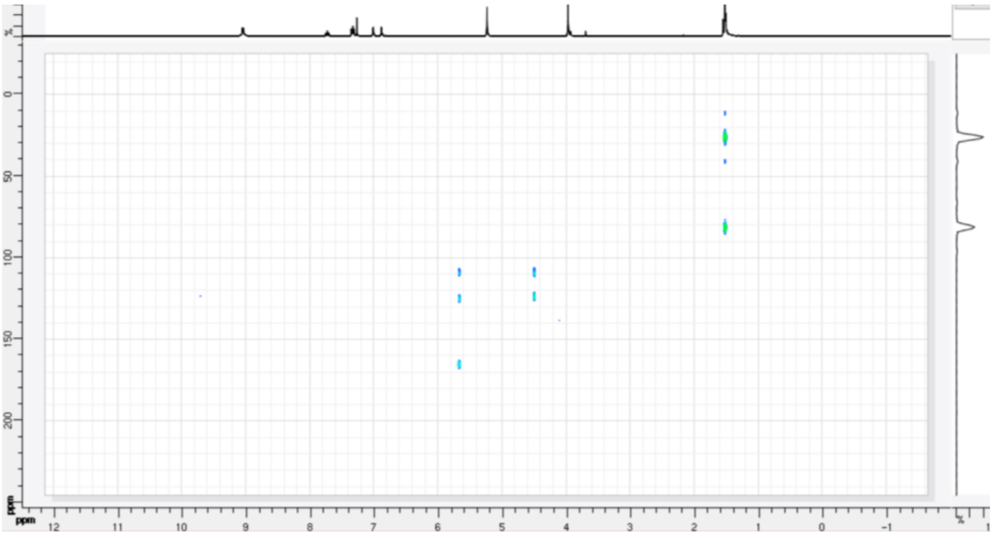

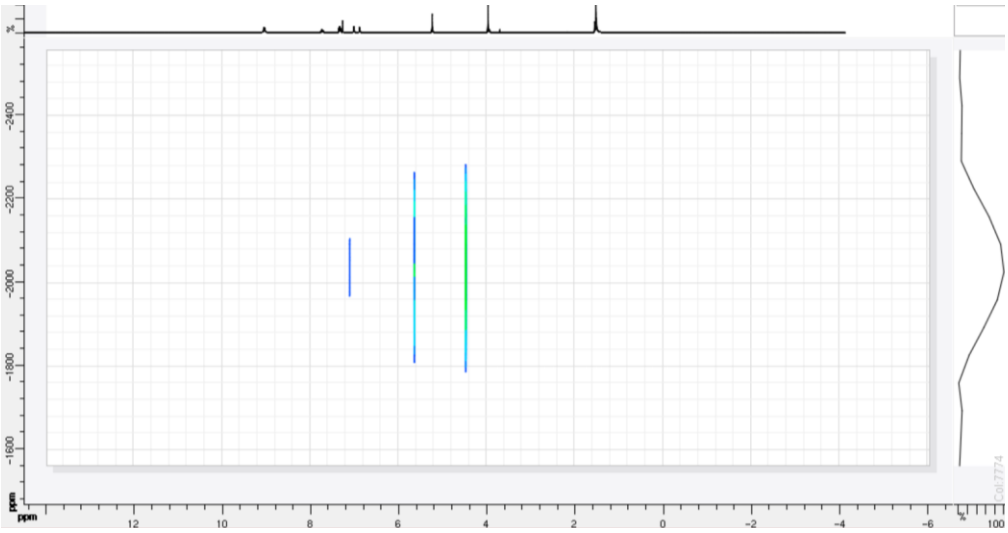

C.10. Characterization of complex 15

15

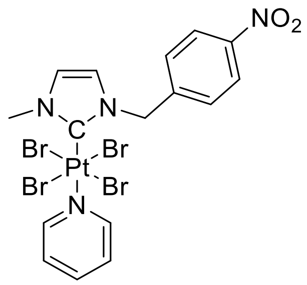

Complex **13** was synthesized according to our reported procedure.[1]

HMQC ^{1}H-^{195}Pt NMR ($CDCl_3$, 64.2 MHz, 20 °C): *δ* – 2067 (m).

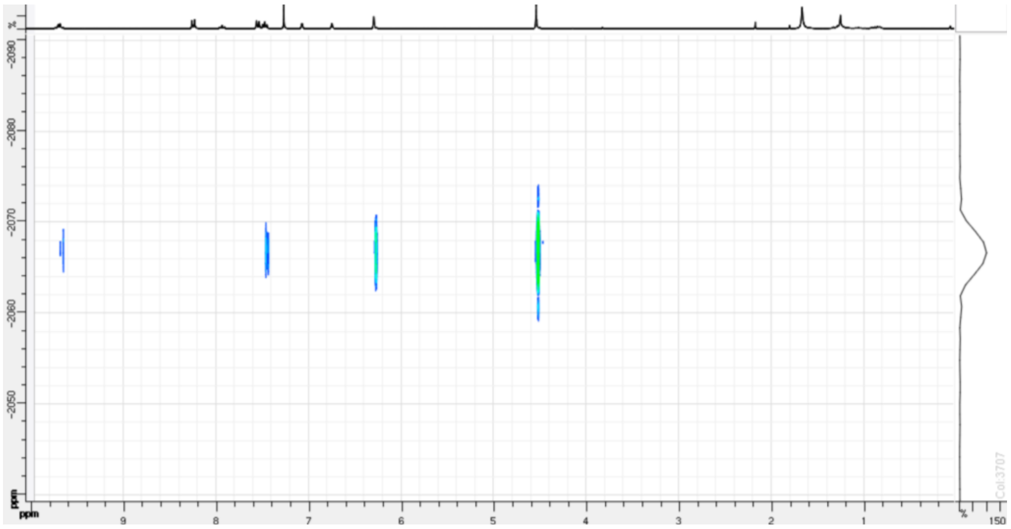

C.11. Characterization of complex 15

16

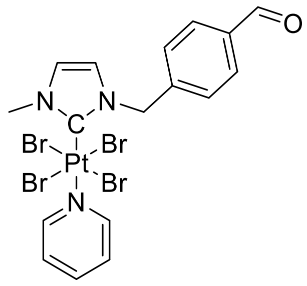

Red solid, 12.3 mg, yield 99%. ^{1}H NMR ($CDCl_3$, 300 MHz, 20 °C): δ 4.50 (s, 3H, N- CH_3), 6.23 (s, 2H, N- CH_2), 6.74 (q, *J1*=4.8 Hz, *J2*=2.5 Hz, 1H, CH_{im}), 7.03 (q, *J1*=4.8 Hz, *J2*=2.5

Hz, 1H, CH_{im}), 7.45 (dd, 2H, H_{ar}), 7.52 (d, 2H, H_{ar}), 7.87-7.94 (m, 3H, H_{pyr}), 9.63-9.72 (m, 2H, H_{pyr}), 10.02 (s, 1H, CHO); ^{13}C NMR ($CDCl_3$, 75 MHz, 20 ℃): δ.3 (N- CH_3), 58.9 (N-CH_2), 110.8 (C-Pt), 124.1 (t, J=10.8Hz, CH_{im}), 124.8 (t, J=18.9Hz, Cpyr), 125.8 (t, J=10.8 Hz, CH_{im}), 129.1 (CH_{ar}), 130.1 (CH_{ar}), 136.2 (Car), 139.5 (Cpyr), 143.0 (Car), 154.4 (Cpyr), 191.6 (CHO); HMQC ^{1}H-^{195}Pt NMR ($CDCl_3$, 64.2 MHz, 20 ℃): δ – 2063 (m).

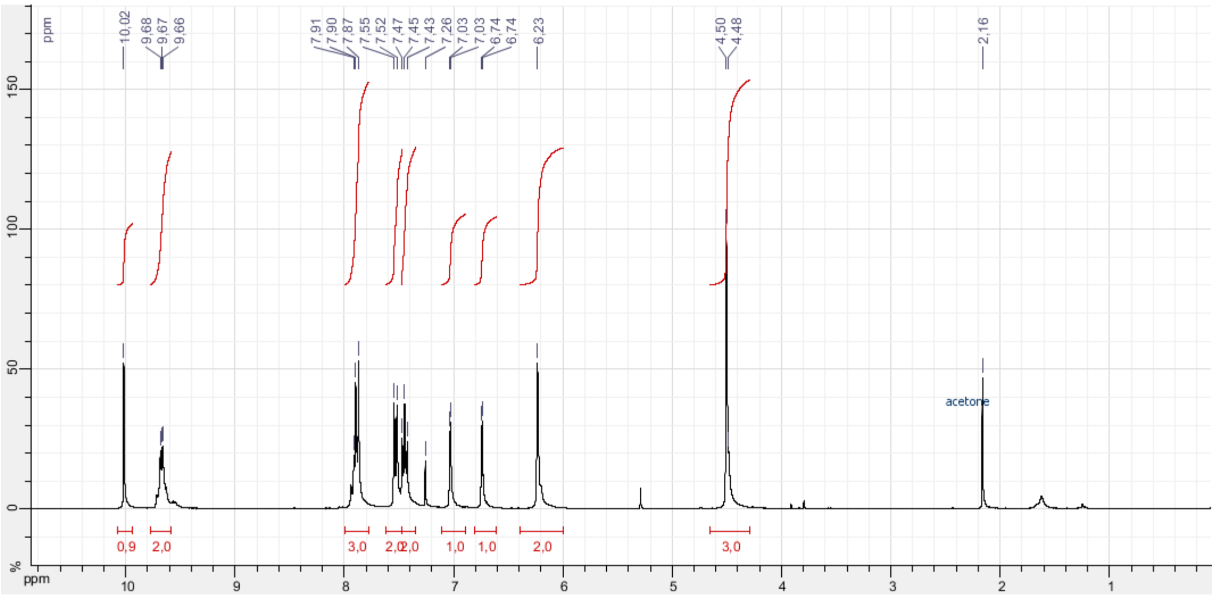

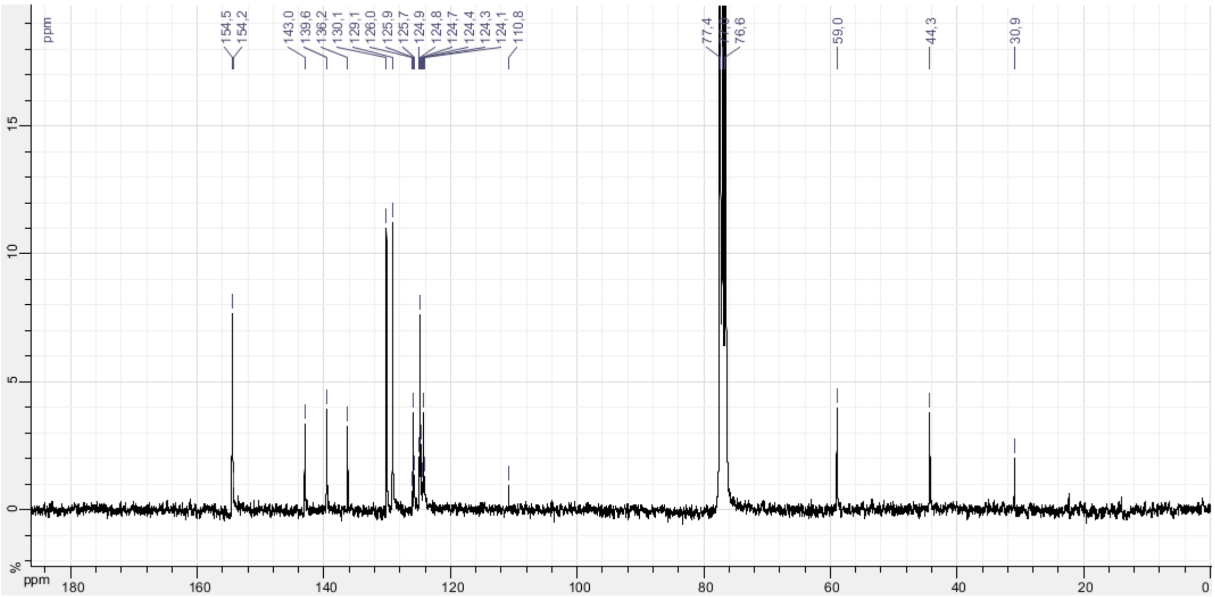

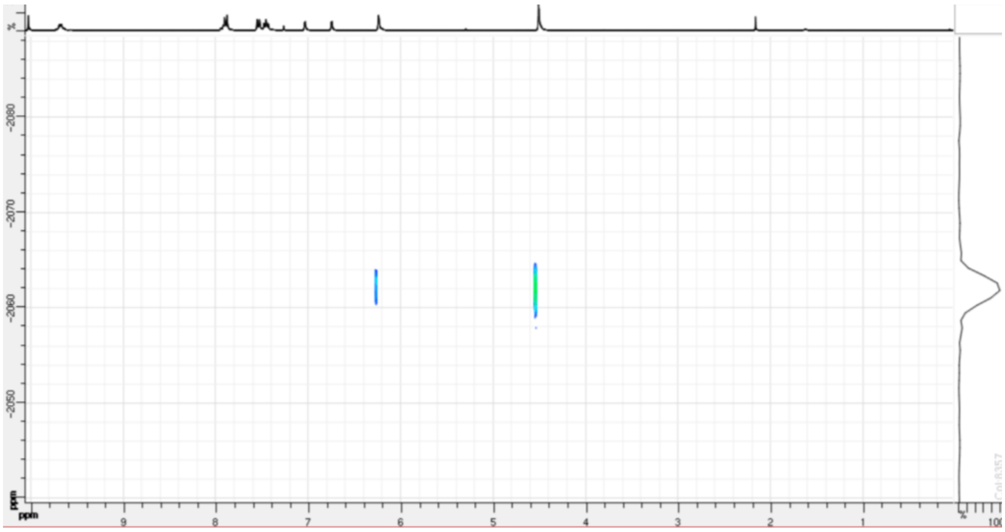

C.12. Characterization of complex 17

17

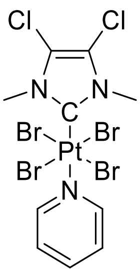

Complex **17** was synthesized according to our reported procedure.[1]

HMQC ^{1}H-^{195}Pt NMR ($CDCl_3$, 64.2 MHz, 20 ℃): δ – 2058 (m).

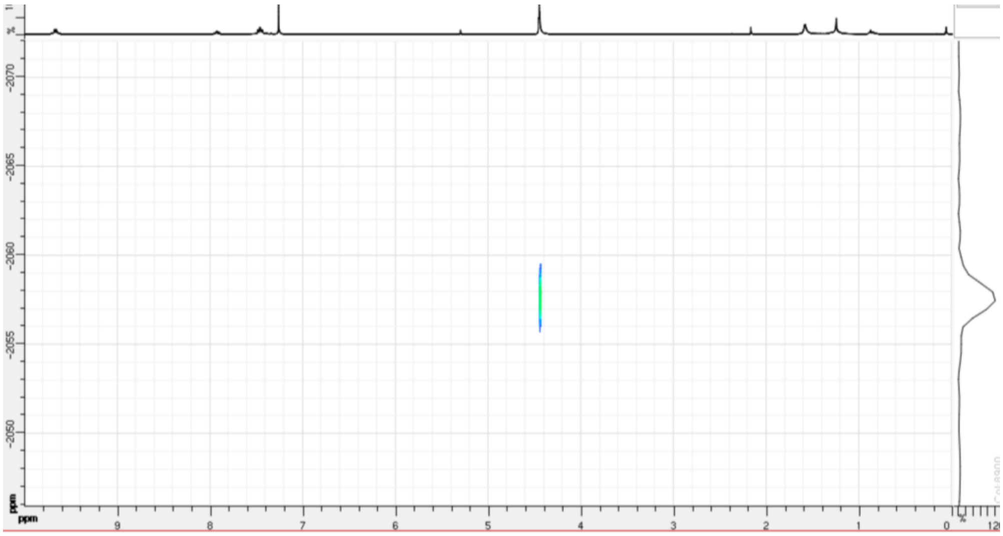

C.13. Characterization of complex 18

18

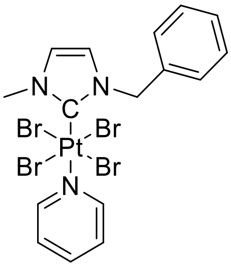

Complex **18** was synthesized according to our reported procedure.[1]

HMQC ^{1}H-^{195}Pt NMR ($CDCl_3$, 64.2 MHz, 20 °C): δ – 2048 (m).

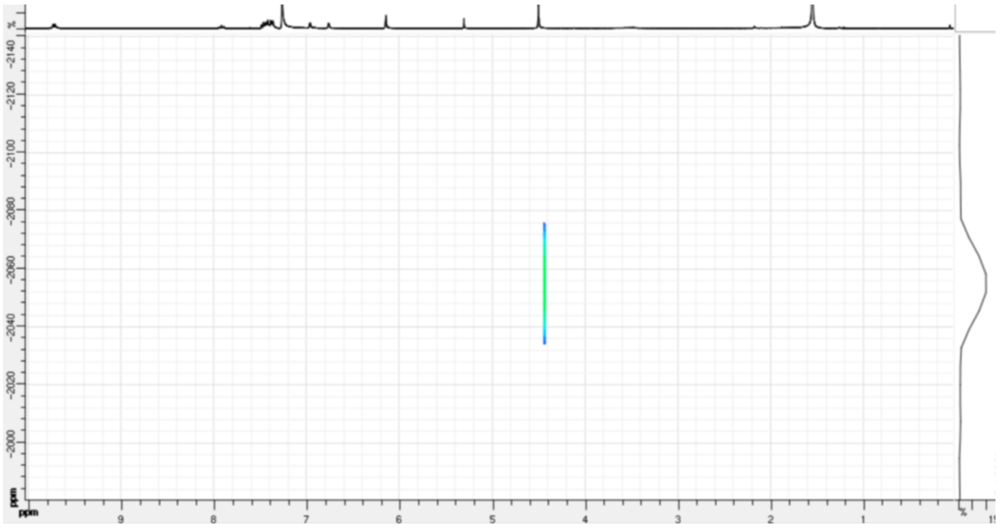

C.14. Characterization of complex 19

19

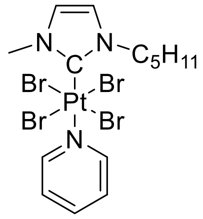

Complex **19** was synthesized according to our reported procedure.[1]

HMQC ^{1}H-^{195}Pt NMR ($CDCl_3$, 64.2 MHz, 20 ℃): δ – 2040 (m).

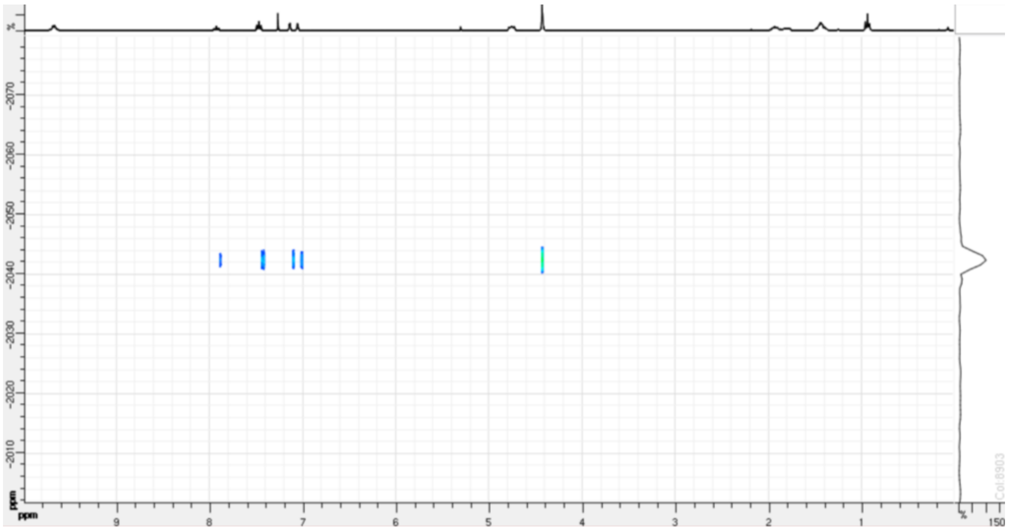

C.15. Characterization of complex 20

20

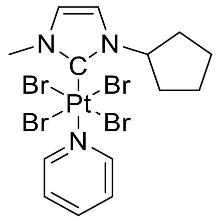

Complex **20** was synthesized according to our reported procedure.[1]

HMQC ^{1}H-^{195}Pt NMR ($CDCl_3$, 64.2 MHz, 20 ℃): δ – 2032 (m).

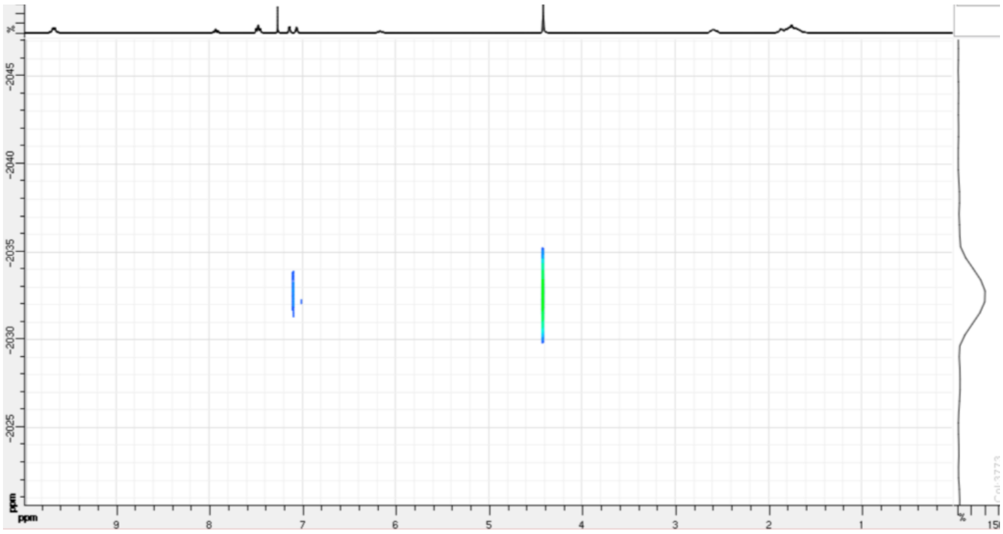

C.16. Characterization of complex 21

21

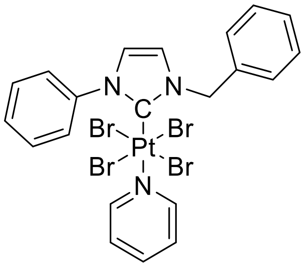

Red solid, 12.2 mg, yield 99%. ^{1}H NMR (CD2Cl2, 500 MHz, 20 ℃): δ 6.38 (s, 2H, N- CH_2), 6.58 (d, *J*=2.0 Hz, 1H, CH_{im}), 7.07 (m, 2H, CH_{ar}), 7.18 (d, *J*=2.0 Hz, 1H, CH_{im}), 7.31 (m, 1H, CH_{ar}), 7.42 (m, 3H, CH_{ar}), 7.60 (m, 5H, CH_{ar}), 8.09 (m, 2H, H_{pyr}), 9.74 (m, 2H, H_{pyr}); ^{13}C NMR (CD2Cl2, 75 MHz, 20 ℃): δ 55.0 (N- CH_2), 114.2 (CH_{im}), 117.2 (CH_{im}), 123.8 (Cpyr), 126.1 (Cpyr), 127.1 (CH_{ar}), 128.9 (CH_{ar}), 129.3 (CH_{ar}), 130.3 (CH_{ar}), 135.8 (C_{ar}), 140.3 (N-C_{ar}), 141.5 (CH_{ar}), 153.7 (C_{pyr}), (C-Pt) not seen; HMQC ^{1}H-^{195}Pt NMR ($CDCl_3$, 64.2 MHz, 20 ℃): δ – 1901 (m); MS (positive ESI) [M - 2Br]: $C_{21}H_{19}Br_2N_3Pt_1$ 667.963, found 667.952.

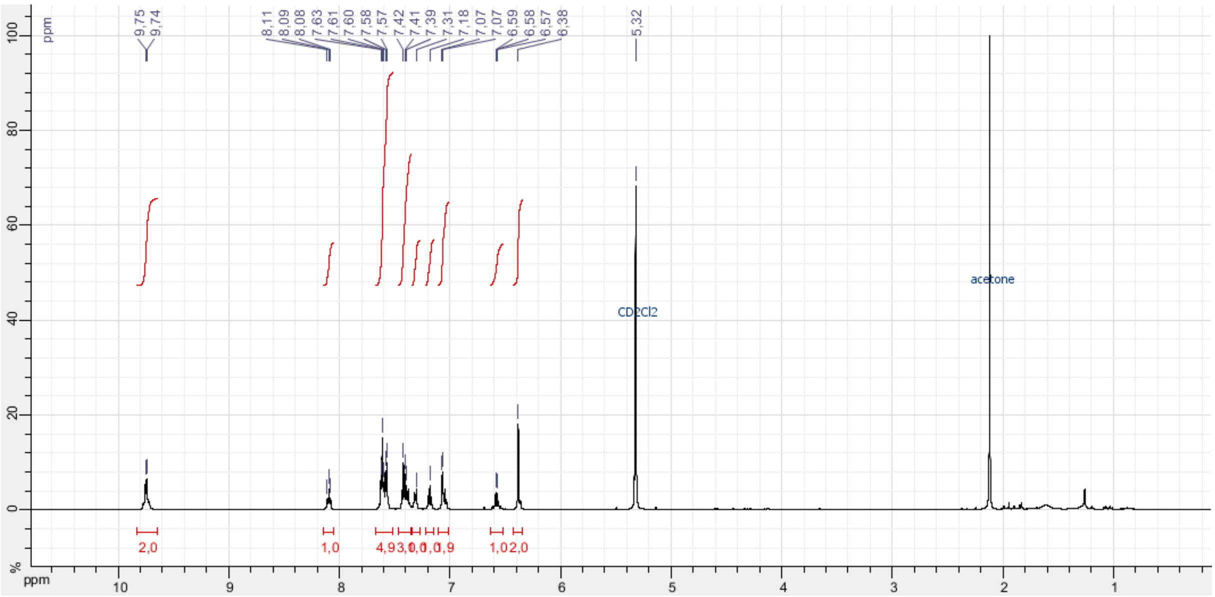
9,75
9,74
8,11
8,09
8,08
7,63
7,61
7,60
7,58
7,57
7,42
7,41
7,39
7,31
7,18
7,07
7,07
6,59
6,58
6,57
6,38
5,32
CD2Cl2
acetone
2,0
1,0
4,93,0,0,1,0,1,9
1,02,0
ppm
%

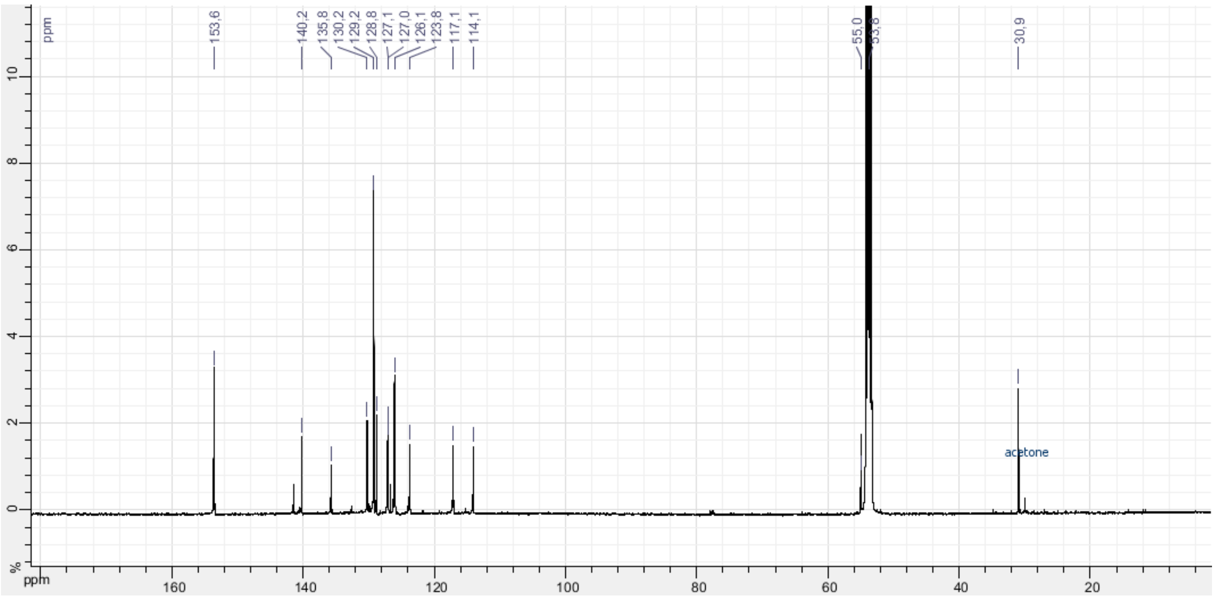
153,6
140,2
135,8
130,2
129,2
128,8
127,1
127,0
126,1
123,8
117,1
114,1
55,0
53,8
30,9
acetone
ppm
%

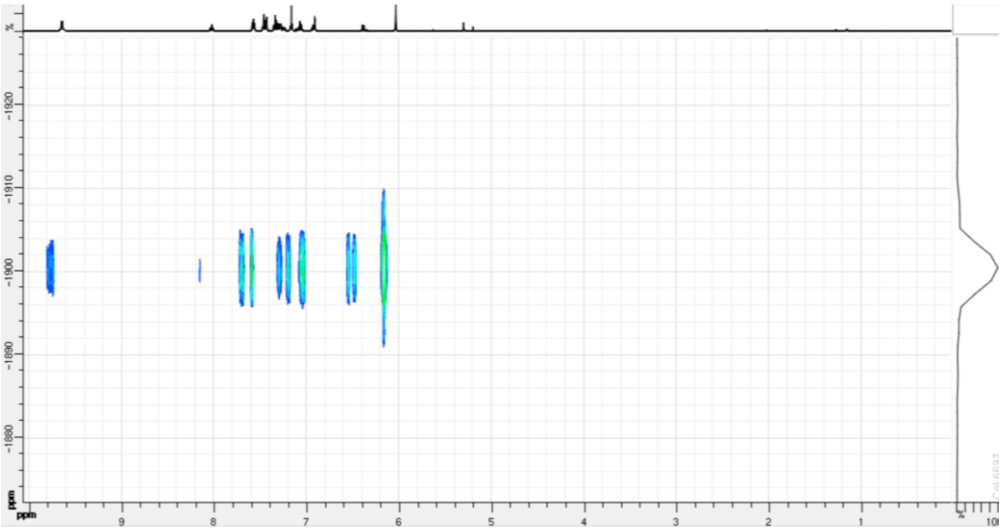
ppm

D) Synthesis of (NHC)PtCl4(amine) complexes

General procedure for the synthesis of (NHC)PtCl4(amine) complexes

In a 10 mL round bottom flask, the precursor (10 mg, 1 equiv.) was dissolved in CH_2Cl_2 (5 mL) and cooled at 0 °C and $PhICl_2$ (10 equiv.) was slowly added. After 1 hour at 0 °C, the addition of pentane (10 mL) caused the precipitation of **22-26** as a light yellow powder, which was filtered off, washed and dried.

D.1. Characterization of complex 22

22

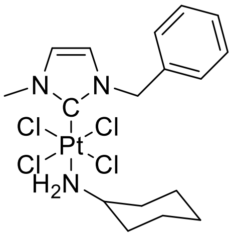

Complex **22** was synthesized according to our reported procedure.[1]

HMQC ^{1}H-^{195}Pt NMR ($CDCl_3$, 64.2 MHz, 20 °C): δ – 883 (m).

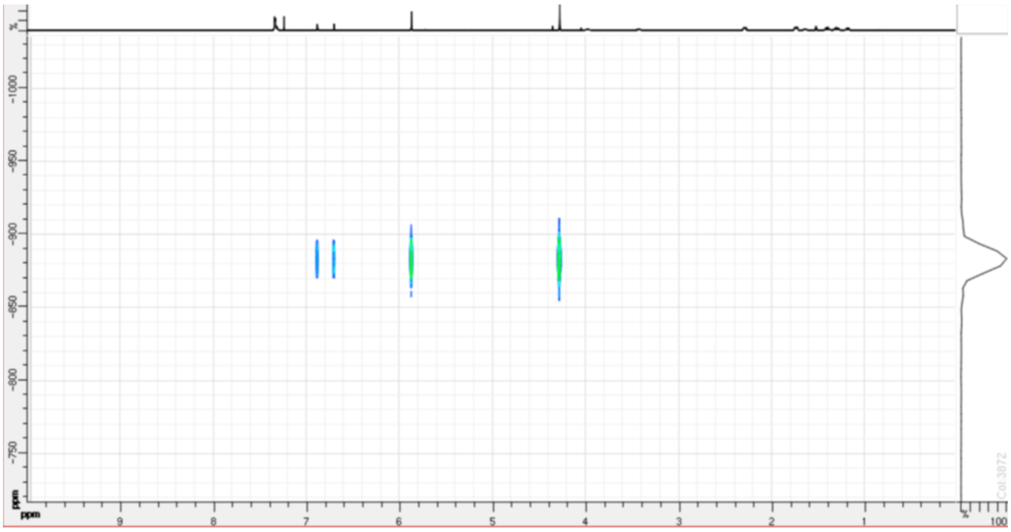

D.2. Characterization of complex 23

23

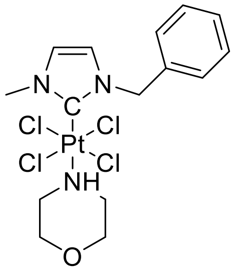

Complex **23** was synthesized according to our reported procedure.[1] HMQC ^{1}H-^{195}Pt NMR ($CDCl_3$, 64.2 MHz, 20 °C): δ – 853 (m).

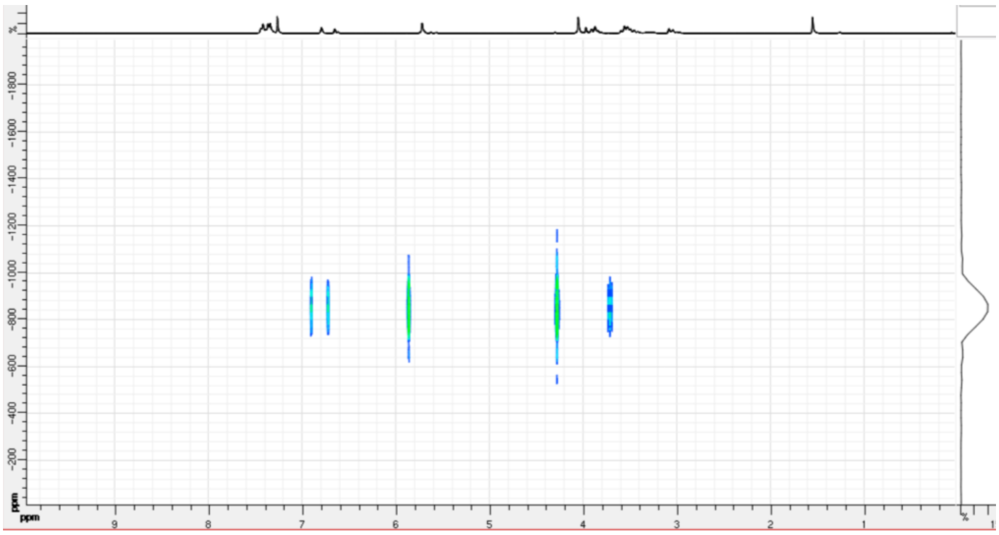

D.3. Characterization of complex 24

24

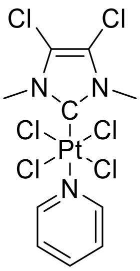

Complex **24** was synthesized according to our reported procedure.[1]

HMQC ^{1}H-^{195}Pt NMR ($CDCl_3$, 64.2 MHz, 20 °C): δ – 825 (m).

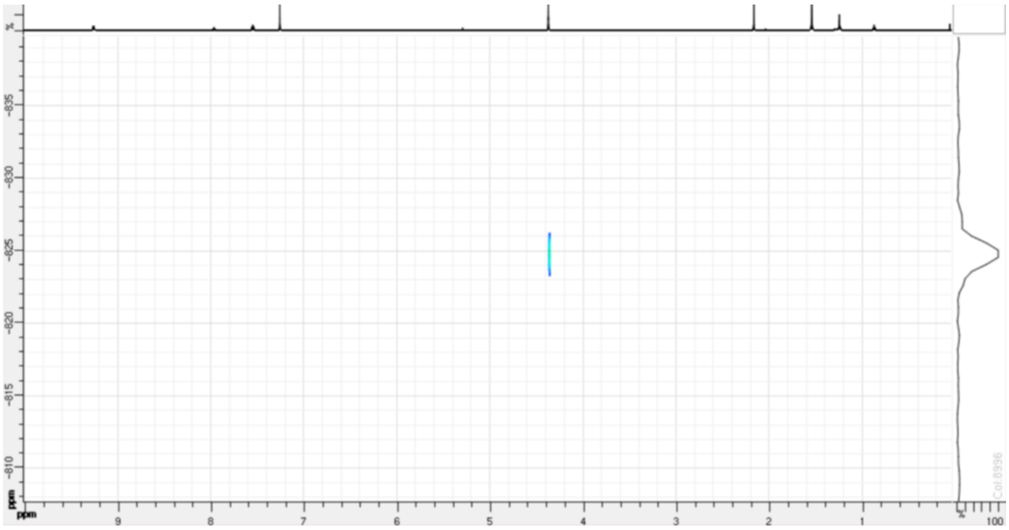

D.4. Characterization of complex 25

25

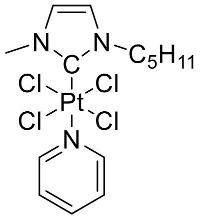

Complex **25** was synthesized according to our reported procedure.

HMQC ^{1}H-^{195}Pt NMR ($CDCl_3$, 64.2 MHz, 20 ℃): δ – 810 (m).

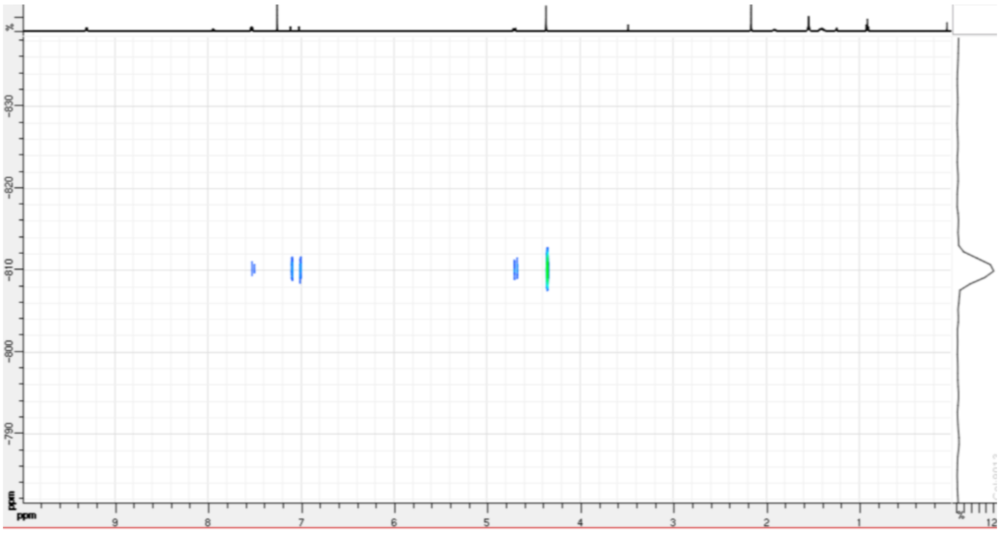

D.5. Characterization of complex 26

26

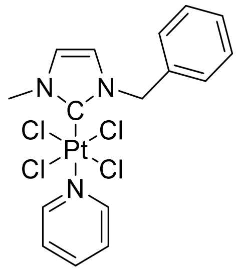

Complex **26** was synthesized according to our reported procedure.[1]

HMQC ^{1}H-^{195}Pt NMR ($CDCl_3$, 64.2 MHz, 20 °C): δ – 795 (m).

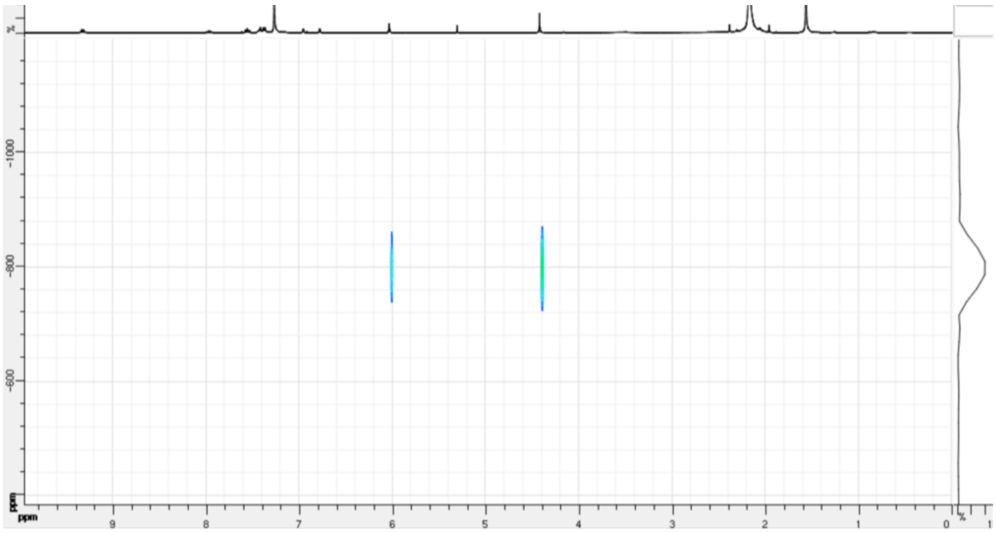

E) Molecular structure of complex 15

**Complexes**	**15**
Empirical formula	C16 H16 Br4 N4 O2 Pt
Formula weight	811.06
Temperature	173(2) K
Wavelength	0.71073 A
Crystal system	*Monoclinic*
space group	*P 21/c*
Unit cell dimensions	
a (Å)	a = 8.3334(3) A
b (Å)	b = 20.2997(9)A
c (Å)	c = 15.0554(4) A
α (°)	alpha = 90 deg
β (°)	beta = 122.400(2) deg
γ (°)	gamma = 90 deg
Volume (Å^3)	2150.38(14) A^3
Z	4
Calculated density (Mg/m^3)	2.505 Mg/m^3
Absorption coefficient (mm^{-1})	13.977 mm^-1
F(000)	1496
Crystal size (mm)	0.28 x 0.15 x 0.12 mm
Theta range (°)	1.890 to 27.501 deg
Limiting indices	-10<=h<=10, -24<=k<=26, -19<=l<=14
Reflections collected / unique / R_{int}	12884 / 4921 [R(int) = 0.0544]
Completeness to theta	100.0 %

Absorption Correction	Semi-empirical from equivalents
Max. and min. transmission	0.11189 and 0.07002
Refinement method	Full-matrix least-squares on F^2
Data / restraints / parameters	4921 / 0 /245
Goodness-of-fit on F_2	1.131
Final R indices *R1, wR2 (I>2σ(I))*	R1 = 0.0358, wR2 = 0.0811
R1, *wR2* (all data)	R1 = 0.0606, wR2 = 0.1187
Largest diff. peak and hole (e.$Å^{-3}$)	1.767 and -2.495 e.A^-3
Extinction coefficient	n/a

[1] a) M. Bouché, P.-A. Bonnefont, T. Achard, S. Bellemin-Laponnaz, Exploring Diversity in Platinum(IV) N-Heterocyclic Carbene Complexes: Synthesis, Characterization, Reactivity and Biological Evaluation, *Dalton Trans.*, **2018**, *47*, 11491-11502; b) M. Bouché, G. Dahm, M. Wantz, S. Fournel, T. Achard, S. Bellemin-Laponnaz, Platinum(IV) N-heterocyclic carbene complexes: their synthesis, characterisation and cytotoxic activity *Dalton Trans*. **2016**, *45*, 11362-11368.

[2] J. K. Muenzner, T. Rehm, B. Biersack, A. Casini, I. A. M. de Graaf, P. Worawutputtapong, A. Noor, R. Kempe, V. Brabec, J. Kasparkova, R. Schobert *J. Med. Chem.* **2015**, *58*, 6283-6292.
